# Effects of Goal Type and Reinforcement Type on Self-Reported Domain-Specific Walking Among Inactive Adults: 2×2 Factorial Randomized Controlled Trial

**DOI:** 10.2196/19863

**Published:** 2020-12-04

**Authors:** Mindy L McEntee, Alison Cantley, Emily Foreman, Vincent Berardi, Christine B. Phillips, Jane C. Hurley, Melbourne F. Hovell, Steven Hooker, Marc A Adams

**Affiliations:** 1 College of Health Solutions Arizona State University Phoenix, AZ United States; 2 Department of Psychology Crean School of Health and Behavioral Sciences Chapman University Orange, CA United States; 3 Atria Senior Living Louisville, KY United States; 4 College of Health and Human Services San Diego State University San Diego, CA United States

**Keywords:** exercise, population health, goals, reward, walking, mHealth, health promotion, health behavior, adaptive intervention, behavioral intervention

## Abstract

**Background:**

WalkIT Arizona was a 2×2 factorial trial examining the effects of goal type (adaptive versus static) and reinforcement type (immediate versus delayed) to increase moderate to vigorous physical activity (MVPA) among insufficiently active adults. The 12-month intervention combined mobile health (mHealth) technology with behavioral strategies to test scalable population-health approaches to increasing MVPA. Self-reported physical activity provided domain-specific information to help contextualize the intervention effects.

**Objective:**

The aim of this study was to report on the secondary outcomes of self-reported walking for transportation and leisure over the course of the 12-month WalkIT intervention.

**Methods:**

A total of 512 participants aged 19 to 60 years (n=330 [64.5%] women; n=425 [83%] Caucasian/white, n=96 [18.8%] Hispanic/Latinx) were randomized into interventions based on type of goals and reinforcements. The International Physical Activity Questionnaire-long form assessed walking for transportation and leisure at baseline, and at 6 months and 12 months of the intervention. Negative binomial hurdle models were used to examine the effects of goal and reinforcement type on (1) odds of reporting any (versus no) walking/week and (2) total reported minutes of walking/week, adjusted for neighborhood walkability and socioeconomic status. Separate analyses were conducted for transportation and leisure walking, using complete cases and multiple imputation.

**Results:**

All intervention groups reported increased walking at 12 months relative to baseline. Effects of the intervention differed by domain: a significant three-way goal by reinforcement by time interaction was observed for total minutes of leisure walking/week, whereas time was the only significant factor that contributed to transportation walking. A sensitivity analysis indicated minimal differences between complete case analysis and multiple imputation.

**Conclusions:**

This study is the first to report differential effects of adaptive versus static goals for self-reported walking by domain. Results support the premise that individual-level PA interventions are domain- and context-specific and may be helpful in guiding further intervention refinement.

**Trial Registration:**

Preregistered at clinicaltrials.gov: (NCT02717663) https://clinicaltrials.gov/ct2/show/NCT02717663

**International Registered Report Identifier (IRRID):**

RR2-10.1016/j.cct.2019.05.001

## Introduction

Few adults meet the recommended physical activity (PA) guidelines, despite evidence of a strong dose-response relationship between PA and a range of health benefits, including decreased mortality [1], improved fitness, improved physical and mental functioning, and enhanced quality of life [2,3]. To date, interventions to increase PA have primarily focused on individual behavior change and had limited impact on population health [4]. Accordingly, there remains a need to develop potent interventions capable of producing an impact on a broader scale.

Mobile health (mHealth) technology provides a platform for increasing intervention reach and quickly tailoring content in response to an individual’s behavior and preferences, but it requires evidence-based and scalable interventions to improve population health. Evidence suggests that behavioral strategies (eg, goal setting, financial reinforcement, feedback on performance) tend to be more effective than cognitive strategies (eg, education, motivation enhancement, self-belief) to increase PA in adults, yet no single strategy has consistently outperformed the rest [1]. It remains unclear how behavioral components may interact with each other in multicomponent interventions.

The WalkIT Arizona trial was designed to address this gap in the literature by studying the effects of an mHealth intervention that combined two evidence-based behavioral strategies—goal setting and positive reinforcement through the use of financial incentives—on objectively measured PA [5]. This paper reports on the study’s secondary outcome: self-reported PA comprised of walking for transportation and leisure, and biking for transportation. Observational research has identified differences in the prevalence and correlates of domain-specific physical activities not otherwise captured via objective measures [6-9]. Thus, we examined intervention effects separately for transportation and leisure and for activity type (walking versus biking). Although studies suggest that participants’ reported PA is less accurate than objective measures [10,11], self-reported data provide additional context that may be useful for understanding participant behavior and guiding the refinement of interventions.

We hypothesized that there would be significant main effects of goal type and reinforcement timing consistent with previous studies: those with adaptive goals would report more PA than those with static goals [12,13], and those receiving immediate reinforcement would report more PA than those receiving delayed reinforcement [13]. We further hypothesized that we would see a combined effect of the two intervention parameters, such that participants receiving adaptive goals and immediate reinforcement would report more walking than all other intervention groups. As the intervention did not specifically target any particular domain or type of PA, we hypothesized that there would be similar effects for transportation and leisure walking, and for transportation biking.

## Methods

### Study Design

WalkIT Arizona was a 2×2 factorial randomized trial evaluating the effects of goal setting (adaptive versus static goals) combined with financial incentives (immediate versus delayed reinforcement) to increase moderate-to-vigorous PA (MVPA) among insufficiently active adults. Participant selection was balanced across geographic information system–measured neighborhood walkability (high/low) and socioeconomic status (high/low) at the census block group level, with recruitment balanced across calendar months to adjust for seasonal effects. These design factors were important to the broader study testing multilevel interactions between individual-level intervention components and neighborhood design factors for PA maintenance during the follow-up period. The study was powered to detect a 2.1 minute/day difference in main effects and 4.2 minute/day difference in interaction effects between groups using accelerometer-measured MVPA, with a sample size of 120 participants per group. Participants completed a 12-month intervention followed by a 12-month observational follow-up period. Self-reported data were collected at baseline and at 6, 12, 18, and 24 months. Analyses presented here were conducted following completion of the 12-month intervention. This study was approved by the local institutional review board; further study details are published elsewhere [5].

### Participants

Insufficiently active adults aged 19 to 60 years (N=512) were randomized for participation between May 2016 and May 2018. Participants were screened online and via phone interview prior to attending an office visit. Inactive status was verified following a 10-day baseline period in which participants were asked to wear a wrist-worn accelerometer during their normal activities. Baseline was extended beyond the scheduled 10 days for some participants due to issues with the accelerometer, problems with the mobile app, nonadherence to accelerometer wear protocol, or illness. Participants were told they would receive one of four different PA interventions. Baseline participant characteristics are displayed in Table 1.

**Table 1:** Baseline participant characteristics.

Characteristics		Total (N=512)	Adaptive goal + immediate reinforcement (n=128)	Static goal + immediate reinforcement (n=128)	Adaptive goal + delayed reinforcement (n=128)	Static goal + delayed reinforcement (n=128)
Age, mean (SD)		45.5 (9.1)	45.6 (9.5)	46.0 (8.9)	46.7 (8.6)	43.5 (9.3)
BMI, mean (SD)		33.9 (7.1)	33.7 (7.3)	33.8 (7.3)	33.6 (7.0)	34.5 (7.6)
Female, n (%)		330 (64.5)	82 (64.1)	80 (62.5)	81 (63.3)	87 (68.0)
**Race and ethnicity^a^, n (%)**						
	White	425 (84.0)	108 (84.4)	106 (82.8)	105 (82.0)	106 (82.8)
	Black	31 (6.1)	5 (3.9)	9 (7.0)	9 (7.0)	8 (6.3)
	American Indian or Alaskan Native	14 (2.7)	4 (3.1)	3 (2.3)	2 (1.6)	5 (3.9)
	Asian	12 (2.3)	4 (3.1)	3 (2.3)	3 (2.3)	2 (1.6)
	Native Hawaiian or other Pacific Islander	7 (1.4)	3 (2.3)	1 (0.8)	2 (1.6)	1 (0.8)
	Hispanic or Latinx	96 (18.8)	22 (17.2)	26 (20.3)	24 (18.8)	24 (18.8)
	Prefer not to answer	32 (6.3)	5 (3.9)	8 (6.3)	10 (7.8)	9 (7.0)
Current tobacco smoker, n (%)		26 (5.1)	3 (2.4)	10 (7.8)	5 (3.9)	8 (6.3)
Current e-smoker, n (%)		10 (2.0)	2 (1.6)	3 (2.4)	1 (0.8)	4 (3.2)
Married/living with partner, n (%)		346 (67.6)	82 (64.1)	85 (66.4)	93 (72.7)	86 (67.2)
**Residence type, n (%)**						
	Single family house	392 (76.6)	94 (73.4)	93 (72.7)	104 (81.3)	101 (78.9)
	Apartment	66 (12.9)	16 (12.5)	18 (14.1)	15 (11.7)	17 (13.3)
Years at current residence, mean (SD)		7.3 (7.4)	7.5 (7.9)	7.3 (7.5)	8.2 (7.0)	6.4 (7.1)
Children residing in household, n (%)		251 (49.0)	61 (47.7)	61 (47.5)	64 (50.0)	65 (50.8)
Number of children in household, mean (SD)		1.0 (1.2)	1.0 (1.2)	1.0 (1.3)	1.0 (1.3)	1.0 (1.1)
Household income, median		$60,000-$79,999	$80,000-$99,999	$60,000-$79,999	$60,000-$79,999	$80,000-$99,999
Education, median		College graduate	College graduate	College graduate	College graduate	College graduate
Employed full time, n (%)		390 (76.2)	98 (76.6)	97 (75.8)	94 (73.4)	101 (78.9)
Distance from home to work (meters), median		16,316	15,368	16,718	15,597	16,926

^a^Race/ethnicity cumulative is greater than 100%, as participants were asked to select all that applied.

### Intervention Components

WalkIT Arizona intervention components have been described in detail elsewhere [5]. Briefly, participants in all four groups were provided with an activity monitor, which they were asked to wear on their wrist for 1 year, and a set of educational materials on the first intervention day. Throughout the intervention phase, participants could receive feedback via text message at any time on their accumulated MVPA minutes once they synced their activity monitor to the automated mHealth servers. All groups also received daily antecedent prompts using a pool of messages from our preliminary studies to evoke motivation, overcome barriers, remind about benefits, and provide other general health advice based on previous research. Text messaging was the primary communication channel between the mHealth system and participants. All feedback, goals, and reinforcement were communicated via this channel. In addition, participants were randomized to receive one of two types of daily goals (adaptive or static) and one of two types of financial incentives (immediate or delayed reinforcement).

#### Goal Setting

Participants allocated to the static goal group were asked to accumulate 30 minutes or more of MVPA daily throughout the 1-year intervention phase (eg, “Goal for 4/1 is 30 minutes”). A static goal of 30 minutes daily on at least 5 days per week aligns with current PA guidelines to obtain 150 minutes/week. Participants allocated to the adaptive goal group were assigned a goal daily based on a previously tested percentile-rank algorithm [12-14]. Unlike a static goal, adaptive goals had the potential to adjust up, down, or stay the same, depending on each participant's unique performance over the previous 9 accelerometer-measured observations (eg, “Goal for 4/1 is 7 minutes”). Each new adaptive goal was valid for the single day only. Regardless of goal type or financial reinforcement timing, participants were praised for meeting their goals (eg, “Well done! Goal met! 32 minutes today…goal for 4/2 is 15 minutes”).

#### Reinforcement Timing

Participants allocated to the delayed, noncontingent reinforcement group received escalating financial reinforcement on a 60-day schedule (ie, US $15 in month 2, US $30 in month 4, US $50 in month 6, US $75 in month 8, and US $95 in month 10) for participating and syncing their accelerometer. Participants allocated to the immediate reinforcement group earned points for meeting PA goals as described elsewhere [5]. For example, “Cheers, James! Goal met! 63 minutes yesterday. Reward points=100! Balance is 400 points. Goal for 7/1 is 35 minutes.” Points were worth US $0.01, and participants in the immediate reinforcement group were sent e-gift cards each time they accumulated US $5.00, since this was the minimum denomination for most gift cards. Participants in both the immediate and delayed reinforcement groups could select from a catalog of available e-gift card retailers (eg, Amazon, Target, Sephora, Home Depot, Walmart, Starbucks, etc) available from Tango Inc and change their selection at any time. E-gift cards were sent to participants using our automated mHealth system that was online 24 hours/day, 365 days per year.

### Measures

Self-reported PA was assessed using sections 2, 4, and 5 of the International Physical Activity Questionnaire (IPAQ)-long form. The IPAQ was part of a larger battery of self-reported measures given at baseline, 6 months, and 12 months. Total self-reported minutes of walking per week were calculated separately for transportation and leisure domains; total self-reported minutes of biking per week were collected for transportation only using IPAQ scoring guidelines. The IPAQ has demonstrated comparable reliability and validity with other self-reported measures of PA [15] and provided an opportunity to examine changes to domain-specific physical activities not otherwise captured via objective measures.

Demographic information was collected via self-report at screening and baseline. Categories for assessing gender, race, and ethnicity were based on National Institutes of Health guidelines. Participants also reported their date of birth, education, marital status, residence type (single family house, apartment), number of adults and children in the household, and years residing at the current address.

### Statistical Analyses

A generalized linear mixed model approach was used to examine intervention effects across treatment groups. Model selection was guided by distributional properties of outcome data. The outcome was total minutes of self-reported PA: time spent walking in the last week was computed separately for transportation and leisure, while time spent biking was limited to the transportation domain. All data distributions were positively skewed, with a relatively large number of zero values at each time point. We determined that hurdle models provided a better conceptual fit than zero-inflation models, as zero values could only result from remaining inactive during the intervention. Hurdle models contain two parts: a binary logit model, or hurdle, which estimated the likelihood of participants reporting 0 minutes/week of activity, and a truncated count regression model, which estimated the total number of reported minutes of activity in the last week (for those reporting values greater than 0). All count models were truncated at zero and used a negative binomial distribution to address overdispersion of data. Separate analyses were used to examine PA by activity type (walking versus biking) and domain (transportation versus leisure).

Negative binomial hurdle (NBH) models provided a nuanced examination of differences across intervention groups by activity type and domain. NBH models tested main effects and interactions among intervention parameters (goal type, reinforcement timing, time) with a random intercept allowed to vary by participant. Models 1 and 2 examined two-way interactions and included the third intervention parameter (ie, goal type or reinforcement timing) as a covariate. Model 3 examined a three-way goal by reinforcement by time interaction but had less power due to the additional interaction term. Time was specified as an ordered factor to allow for examination of linear and quadratic trends over the course of the intervention, as intervention components were assumed to have nonlinear effects at the individual level. All models were adjusted for census block-level socioeconomic status and neighborhood walkability since these factors were part of the broader research design. Predictor variables were kept consistent for hurdle and count models. All models were estimated using the generalized linear mixed models using template model builder (glmmTMB) package in R [16] and utilized the truncated_nbinom2 family for error distribution.

As glmmTMB models are estimated using only complete cases, we conducted a sensitivity analysis to examine the impact of missing data. Original analyses were compared to models estimated using (1) baseline values carried forward and (2) multiple imputation. Multiple imputation was performed using the multivariate imputation by chained equations (MICE) package [17] with 12 iterations, corresponding to the percentage of missing data [18]. Effects were pooled separately for count and hurdle models. The effects package [19,20] was used to visualize interactions.

## Results

**Figure 1:**
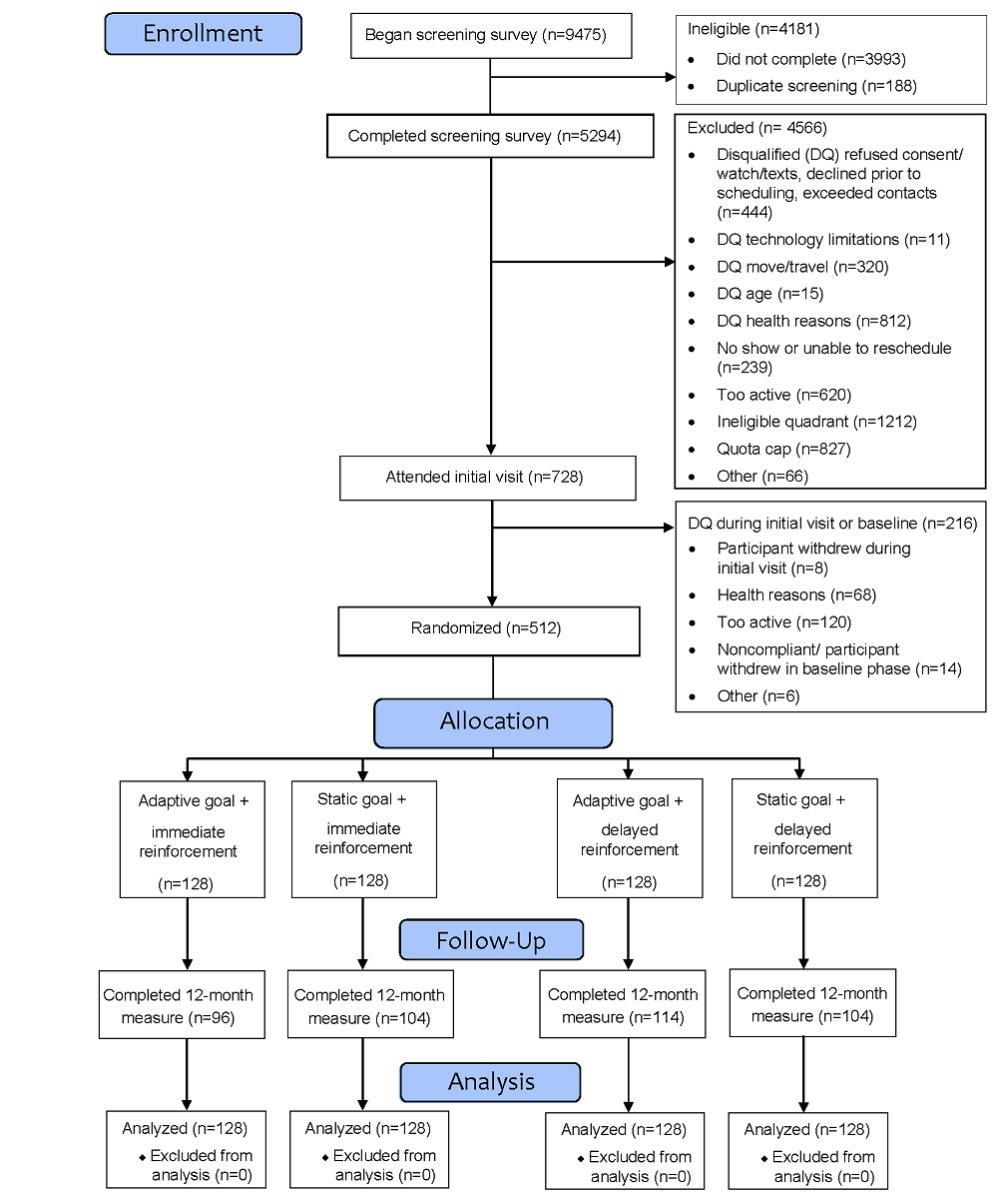
Participant flow.

**Figure 2:**
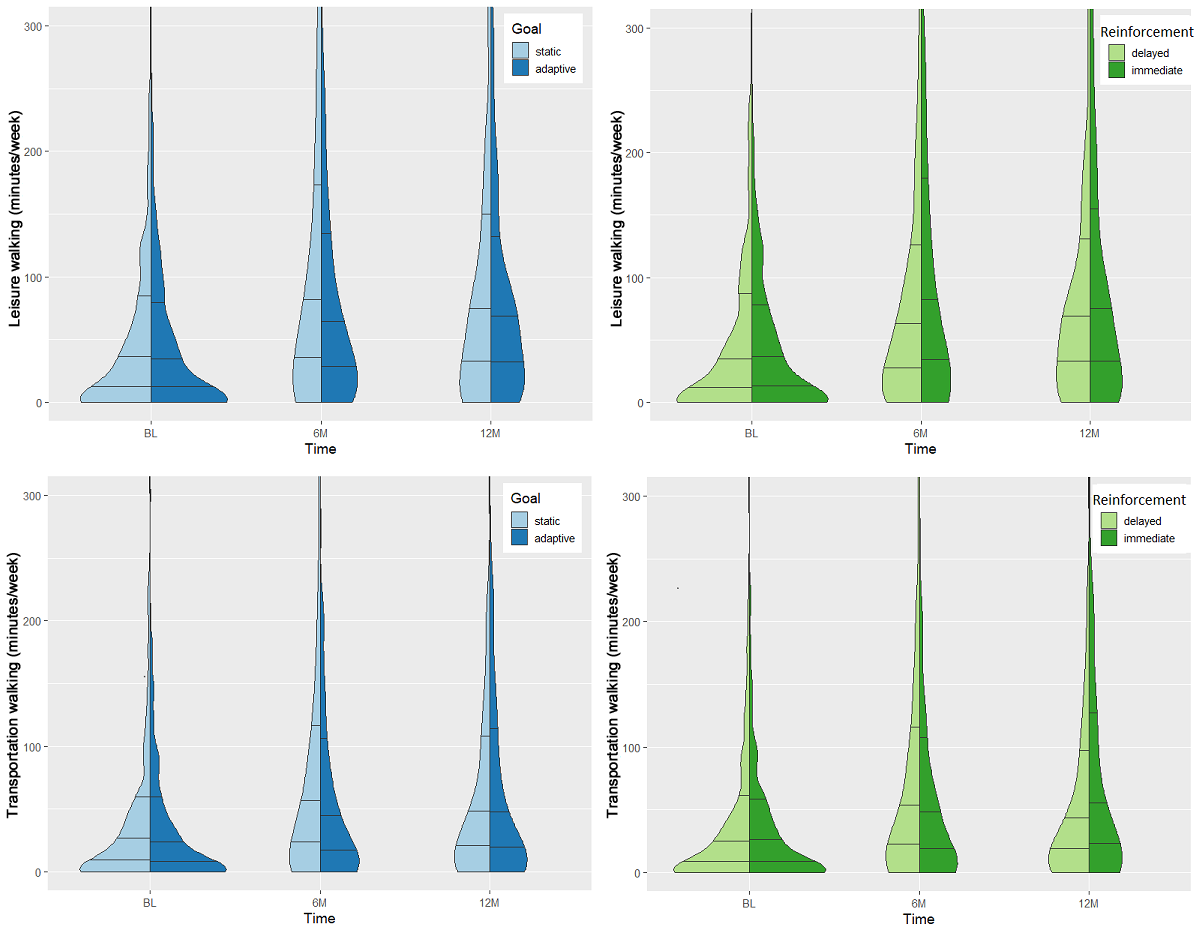
Split violin plots showing distribution of self-reported walking at baseline (BL), 6 months (6M), and 12 months (12M). Horizontal lines indicate 25th, 50th, and 75th percentiles computed from density estimates.

Participant flow is depicted in Figure 1. Participants reported a similar pattern of unadjusted walking activity across intervention groups: both leisure and transportation walking time increased from baseline to 6 months, then decreased from 6 months to 12 months. Across groups, reported walking time at 12 months remained greater than baseline for both leisure (*F*_1.9,764.5_=35.79; *P*<.001; partial eta-square=0.08) and transportation (*F*_1.6,653.7_=15.63; *P*<.001; partial eta-square=0.04). Biking for transportation was reported by a small subset of participants at each time point. Mean reported transportation biking for the entire sample showed a nonsignificant increase from baseline to 12 months; the trajectory of unadjusted transportation biking varied by intervention group. Participants with at least 1 missing self-reported PA data point (121/512, 23.6%) were more likely to report living with a partner but did not significantly differ from those with complete data on any other demographic characteristic. Split violin plots comparing the distribution of self-reported walking time by goal and reinforcement type are depicted in Figure 2. Mean self-reported PA times by intervention group, activity type, and domain are shown in Table 2.

**Table 2:** Self-reported leisure walking, transportation walking, and transportation biking (minutes/week).

Self-reported physical activity			Total (N=512)	Adaptive goal + immediate reinforcement (n=128)	Static goal + immediate reinforcement (n=128)	Adaptive goal + delayed reinforcement (n=128)	Static goal + delayed reinforcement (n=128)
**Leisure walking**							
	Baseline						
		Mean^a^ (SD)	45.3 (77.0)	41.1 (93.5)	36.6 (61.7)	37.9 (87.6)	53.3 (115.0)
		Reported >0 min^b^, n (%)^c^	296 (57.8)	73 (57.0)	75 (58.6)	70 (54.7)	78 (60.9)
	6 months						
		Mean (SD)	97.3 (128.2)	78.7 (155.2)	102.6 (196.2)	80.1 (170.6)	80.1 (100.8)
		Reported >0 min, n (%)	331 (76.6)	80 (20.0)	79 (71.2)	91 (80.5)	81 (75.0)
	12 months						
		Mean (SD)	89.8 (110.3)	78.0 (113.0)	65.7 (87.9)	58.5 (92.4)	58.0 (78.7)
		Reported >0 min, n (%)	329 (78.7)	76 (79.2)	79 (76.0)	95 (83.3)	79 (76.0)
**Transportation walking**							
	Baseline						
		Mean (SD)	42.2 (91.4)	40.2 (51.5)	38.0 (48.9)	39.4 (62.1)	63.4 (120.6)
		Reported >0 min, n (%)	297 (58.0)	76 (59.4)	73 (57.0)	67 (52.3)	81 (63.3)
	6 months						
		Mean (SD)	85.5 (160.0)	93.2 (113.5)	117.6 (155.7)	92.9 (126.2)	84.9 (110.0)
		Reported >0 min, n (%)	305 (70.0)	67 (66.3)	80 (71.4)	77 (67.0)	81 (75.0)
	12 months						
		Mean (SD)	64.6 (93.4)	83.8 (95.6)	102.2 (133.1)	86.8 (102.2)	86.4 (106.9)
		Reported >0 min, n (%)	296 (70.6)	67 (69.8)	74 (71.2)	78 (67.8)	77 (74.0)
**Transportation biking**							
	Baseline						
		Mean (SD)	4.6 (25.8)	9.3 (43.1)	4.9 (23.2)	2.9 (11.1)	1.0 (9.4)
		Reported >0 min, n (%)	33 (6.4)	12 (9.1)	9 (6.9)	9 (7.0)	3 (2.3)
	6 months						
		Mean (SD)	5.4 (29.2)	5.5 (22.8)	4.2 (22.9)	4.5 (18.6)	7.7 (45.5)
		Reported >0 min, n (%)	33 (7.5)	11 (10.6)	6 (5.3)	10 (8.7)	6 (5.6)
	12 months						
		Mean (SD)	5.6 (25.7)	3.9 (17.0)	2.6 (10.8)	9.9 (35.8)	5.4 (29.5)
		Reported >0 min, n (%)	34 (8.0)	6 (6.1)	9 (8.5)	13 (11.3)	6 (5.8)

^a^Calculated means include respondents reporting no physical activity (ie, 0 minutes).

^b^min: minutes.

^c^Percentages are based on total number of participant responses received for each time point.

The subheadings below indicate the interaction specified in NBH models, with other remaining parameters entered as covariates. As sensitivity analysis revealed little impact of missing data, the results discussed below are for complete cases; model parameters using multiple imputation are presented in Multimedia Appendices 1 to 6. Any differences between complete case analysis and multiple imputation results are noted below.

### Leisure Walking

#### Model 1: Goal by Time

The overall proportion of participants reporting any (versus no) leisure walking increased from 57.8% (296/512) at baseline to 78.7% (329/418) at 12 months, as shown in Table 2. NBH model results are displayed as odds and risk ratios in Multimedia Appendix 7. For the hurdle model, participants with static goals had greater odds of reporting any (versus no) leisure walking at baseline, whereas those with adaptive goals had greater odds of reporting any (versus no) leisure walking at 6 and 12 months, although this interaction was nonsignificant (*P*=.08). For the count model, consisting only of participants who reported leisure walking, those who received adaptive goals reported 17% fewer minutes of leisure walking compared to those with static goals (95% CI 0.72-0.95). Lack of a significant goal by time interaction indicated that leisure walking did not differ by goal type across time points.

#### Model 2: Reinforcement by Time

**Figure 3:**
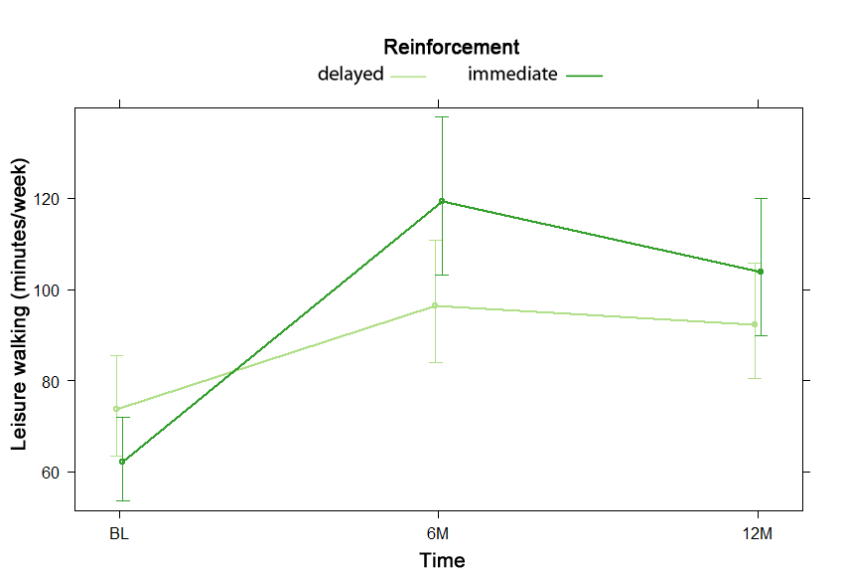
Reinforcement by time interaction in negative binomial count model 2 for leisure walking at baseline (BL), 6 months (6M), and 12 months (12M).

NBH model results displaying odds and risk ratios are shown in Multimedia Appendix 8. For the hurdle model, there was no difference in the odds of reporting any (versus no) leisure walking based on reinforcement type, nor was there any significant reinforcement by time interaction effect. For the count model, consisting only of participants who reported leisure walking, there was a significant reinforcement by time interaction, indicating both linear (risk ratio [RR]=1.22, 95% CI 1.02-1.46) and quadratic (RR=0.79, 95% CI 0.67-0.94) effects. As shown in Figure 3, while all participants reported increased leisure walking time from baseline to 6 months, this increase was greater among participants who received immediate compared with delayed reinforcement. From 6 to 12 months, those with immediate reinforcement reported a decrease in time spent walking for leisure, whereas those with static reinforcement reported a very slight increase.

#### Model 3: Goal by Reinforcement by Time

**Figure 4:**
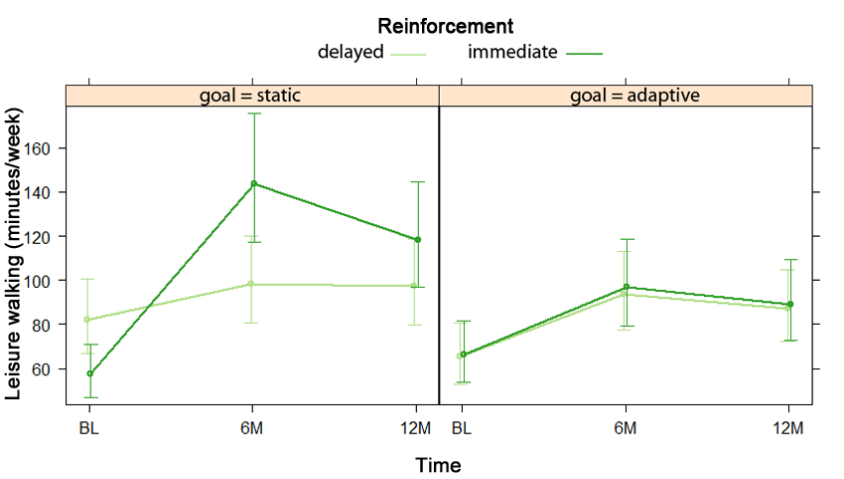
Goal by reinforcement by time interaction in negative binomial count model 3 for leisure walking at baseline (BL), 6 months (6M), and 12 months (12M).

Conditional estimates for an NBH model testing a three-way goal by reinforcement by time interaction and nested two-way interactions for leisure walking are displayed in Table 3. For the hurdle model, the results mirrored model 1: participants with static goals had greater odds of reporting any leisure walking at baseline, whereas those with adaptive goals had greater odds of reporting any leisure walking at 6 and 12 months, although this goal by time interaction was nonsignificant (*P*=.07). For the count model, which consisted only of participants who endorsed leisure walking, the mean reported time was 96.84 minutes/week (95% CI 81.65-114.84). Figure 4 shows a significant three-way interaction in which the reinforcement timing by linear time interaction effect varied significantly by goal type (RR=0.68, 95% CI 0.48-0.97).

**Table 3:** Negative binomial hurdle model examining goal by reinforcement by time (model 3) for leisure walking.

	Zero hurdle model		Count model	
Parameter^a^	OR^b,c^ (95% CI)	*P* value	RR^d^ (95% CI)	*P* value
Intercept	3.45 (2.30-5.18)	<.001****	96.84 (81.65-114.84)	<.001****
Socioeconomic status block (high)	0.82 (0.60-1.12)	.165	0.89 (0.77-1.02)	.131
Walkability block (high)	0.94 (0.69-1.29)	.641	1.03 (0.90-1.18)	.637
Goal (adaptive)	1.15 (0.74-1.80)	.540	0.84 (0.69-1.01)	.067*
Reinforcement (immediate)	0.88 (0.57-1.37)	.512	1.07 (0.88-1.30)	.580
Time: linear	1.80 (1.15-2.81)	.009***	1.13 (0.94-1.34)	.184
Time: quadratic	0.70 (0.43-1.11)	.129	0.92 (0.78-1.10)	.368
Goal by time: linear	1.79 (0.95-3.39)	.073*	1.09 (0.85-1.40)	.488
Goal by time: quadratic	0.90 (0.46-1.79)	.771	1.00 (0.79-1.28)	.987
Reinforcement by time: linear	1.07 (0.57-2.02)	.875	1.47 (1.15-1.89)	.002***
Reinforcement by time: quadratic	1.14 (0.60-2.20)	.678	0.70 (0.55-0.90)	.005***
Goal by reinforcement	1.07 (0.57-2.00)	.746	1.01 (0.76-1.32)	.842
Goal by reinforcement by time: linear	0.69 (0.28-1.71)	.465	0.68 (0.48-0.97)	.033**
Goal by reinforcement by time: quadratic	0.80 (0.31-2.08)	.711	1.28 (0.90-1.81)	.156

^a^Referent groups for parameters are listed in parentheses.

^b^Odds ratio (OR) reflects the odds of reporting any leisure walking (versus none).

^c^OR, risk ratio (RR), and 95% CI are exponentiated coefficients of conditional estimates.

^d^RR reflects the proportional increase (values >1) or decrease (values <1) in non-zero leisure walking minutes/week associated with a one unit change in the predictor.

*P<.1

**P<.05,

***P<.01,

****P<.001.

### Transportation Walking

#### Model 1: Goal by Time

The overall proportion of participants reporting any (versus no) transportation walking increased from 58.0% (297/512) at baseline to 70.6% (296/419) at 12 months, as shown in Table 2. NBH model results are displayed as odds and risk ratios in Multimedia Appendix 9. For the hurdle model, there was no significant difference in odds of reporting any transportation walking by goal type, nor were there any significant goal by time interaction effects. For the count model, which consisted only of participants who reported transportation walking, there were no significant differences in reported transportation walking time by goal type, nor were there any significant goal by time interaction effects.

#### Model 2: Reinforcement by Time

**Figure 5:**
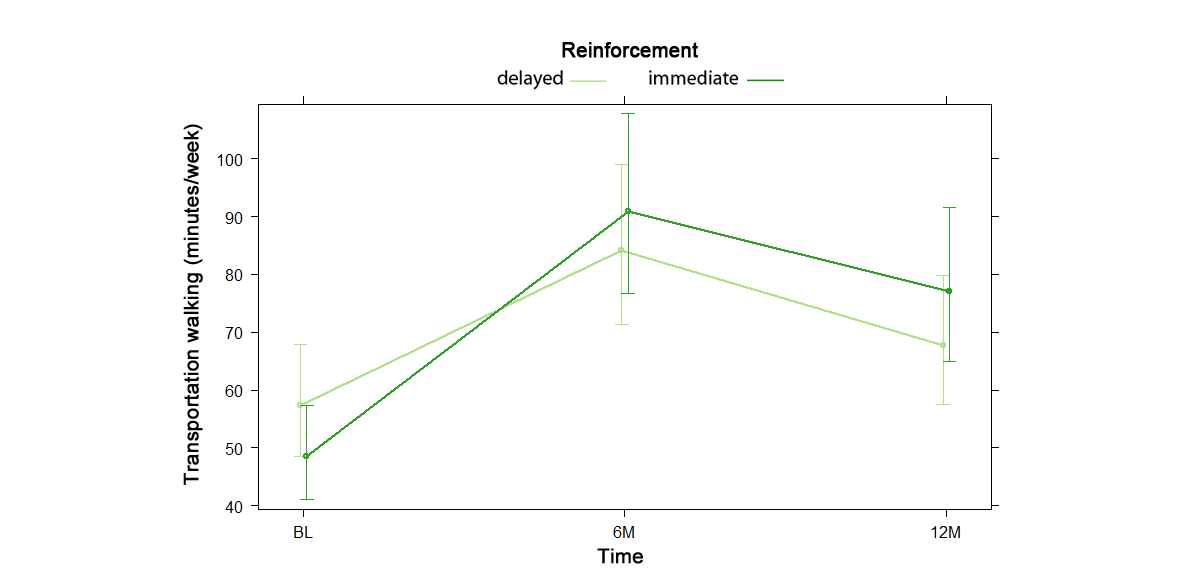
Reinforcement by time interaction in negative binomial count model 2 for transportation walking at baseline (BL), 6 months (6M), and 12 months (12M).

NBH model results are shown as odds and risk ratios in Multimedia Appendix 10. For the hurdle model, there was no significant difference in odds of reporting any transportation walking by reinforcement type, nor was there any significant reinforcement by time interaction effect. For the count model, which consisted only of participants who endorsed transportation walking, time spent walking for transportation did not differ by reinforcement type. As shown in Figure 5, there was a significant reinforcement by time interaction such that participants with immediate reinforcements outperformed those with delayed reinforcements, with a greater increase in transportation walking from baseline to 6 months, and a smaller decrease from 6 to 12 months. However, this effect was no longer significant in multiple imputation analysis (*P*=.07).

#### Model 3: Goal by Reinforcement by Time

Conditional estimates for an NBH model testing a three-way goal by reinforcement by time interaction and nested two-way interactions for transportation walking are shown in Table 4. For the hurdle model, there were no independent or interaction effects for goal type or reinforcement timing on odds of reporting any (versus no) transportation walking. Among participants who endorsed transportation walking in the count model, mean reported time was 81.98 minutes/week (95% CI 66.04-101.77). There were no significant independent or interaction effects for goal type or reinforcement timing on reported transportation walking minutes/week.

**Table 4:** Negative binomial hurdle model examining goal by reinforcement by time (model 3) for transportation walking.

	Zero hurdle model		Count model	
Parameter^a^	OR^b,c^ (95% CI)	*P* value	RR^d^ (95% CI)	*P* value
Intercept	3.00 (1.80-4.98)	<.001***	81.98 (66.04-101.77)	<.001***
Socioeconomic status block (high)	0.79 (0.54-1.17)	.240	0.70 (0.59-0.83)	<.001***
Walkability block (high)	1.83 (1.23-2.71)	.003**	1.03 (0.86-1.22)	.711
Goal (adaptive)	0.65 (0.37-1.13)	.132	0.99 (0.77-1.26)	.912
Reinforcement (immediate)	0.81 (0.46-1.43)	.417	1.00 (0.79-1.28)	.956
Time: linear	1.67 (1.03-2.72)	.038*	1.07 (0.89-1.29)	.450
Time: quadratic	0.75 (0.45-1.25)	.270	0.75 (0.63-0.91)	.003**
Goal by time: linear	1.24 (0.64-2.41)	.526	1.10 (0.84-1.44)	.489
Goal by time: quadratic	0.81 (0.40-1.64)	.557	1.12 (0.86-1.47)	.405
Reinforcement by time: linear	1.13 (0.58-2.22	.745	1.22 (0.93-1.59)	.120
Reinforcement by time: quadratic	0.79 (0.39-1.63)	.545	0.95 (0.72-1.24)	.687
Goal by reinforcement	1.39 (0.63-3.06)	.299	1.03 (0.69-1.38)	.891
Goal by reinforcement by time: linear	0.67 (0.26-1.75)	.416	1.01 (0.69-1.49)	.881
Goal by reinforcement by time: quadratic	1.72 (0.63-4.70)	.292	1.07 (0.73-1.57)	.735

^a^Referent groups for parameters are listed in parentheses.

^b^Odds ratio (OR) reflects the odds of reporting any leisure walking (versus none).

^c^OR, risk ratio (RR), and 95% CI are exponentiated coefficients of conditional estimates.

^d^RR reflects the proportional increase (values >1) or decrease (values <1) in non-zero transportation walking minutes/week associated with a one unit change in the predictor.

*P<.05.

**P<.01.

***P<.001.

### Transportation Biking

#### Model 1: Goal by Time

**Figure 6:**
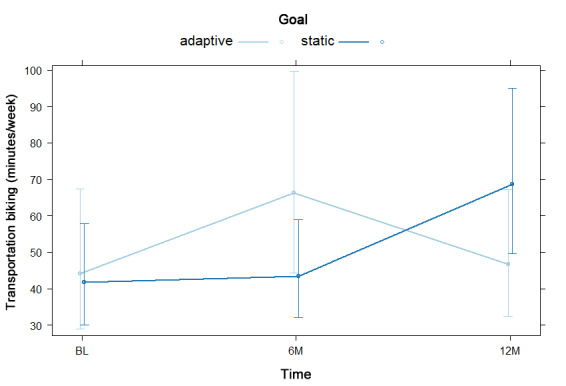
Goal by time interaction in negative binomial count model 1 for transportation biking at baseline (BL), 6 months (6M), and 12 months (12M).

The overall proportion of participants reporting any (versus no) transportation biking increased from 6.4% (33/512) at baseline to 8.0% (34/425) at 12 months, as shown in Table 2. NBH model results are displayed as odds and risk ratios in Multimedia Appendix 11. For the hurdle model, there was no significant difference in odds of reporting any transportation biking by goal type, nor was there any significant goal by time interaction effect. For the count model, which consisted only of participants who reported transportation biking, there was no significant difference in reported transportation biking time by goal type. There was a significant goal by time interaction such that participants with static goals reported more transportation biking than those with adaptive goals at baseline and 6 months, whereas participants with adaptive goals reported more transportation biking than those with static goals at 12 months, as shown in Figure 6.

#### Model 2: Reinforcement by Time

**Figure 7:**
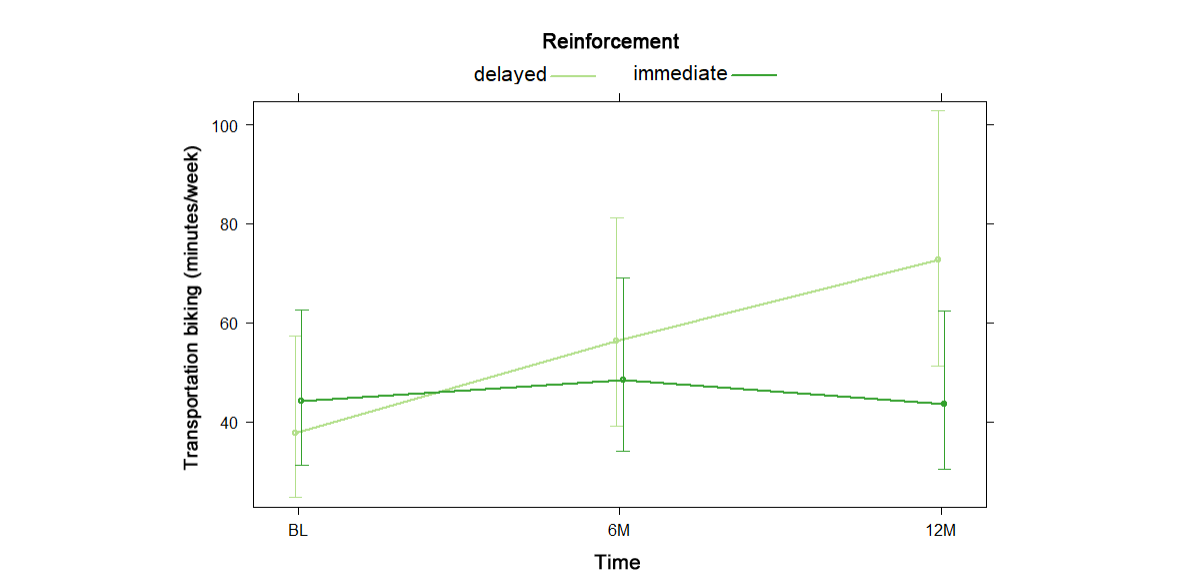
Reinforcement by time interaction in negative binomial count model 2 for transportation biking at baseline (BL), 6 months (6M), and 12 months (12M).

NBH model results are shown as odds and risk ratios in Multimedia Appendix 12. For the hurdle model, there was no significant difference in odds of reporting any transportation biking by reinforcement type, nor was there any significant reinforcement by time interaction effect. For the count model, which consisted only of participants who endorsed transportation biking, there was no significant difference in transportation biking by reinforcement type. There was a significant reinforcement by time interaction such that participants with immediate reinforcement reported more transportation biking than those with delayed reinforcement at baseline, whereas participants with delayed reinforcement reported more transportation biking than those with immediate reinforcement at 6 and 12 months, as shown in Figure 7.

#### Model 3: Goal by Reinforcement by Time

**Figure 8:**
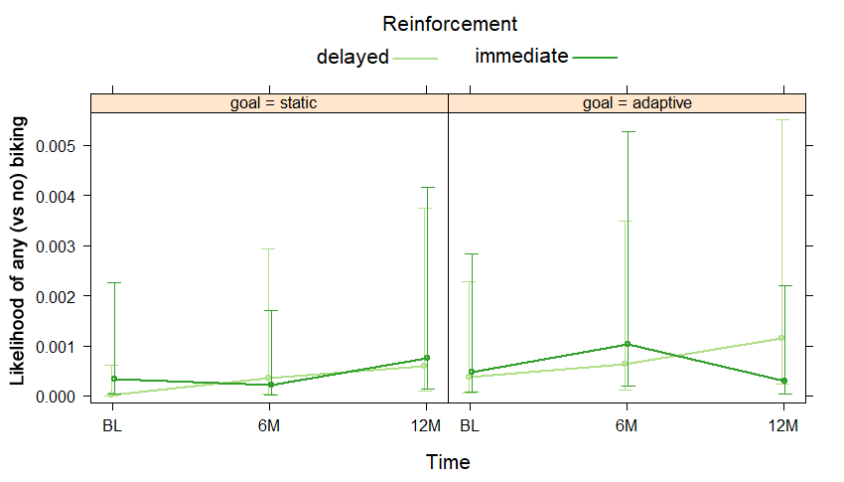
Goal by reinforcement by time interaction for negative binomial hurdle model 3 for transportation biking at baseline (BL), 6 months (6M), and 12 months (12M).

**Figure 9:**
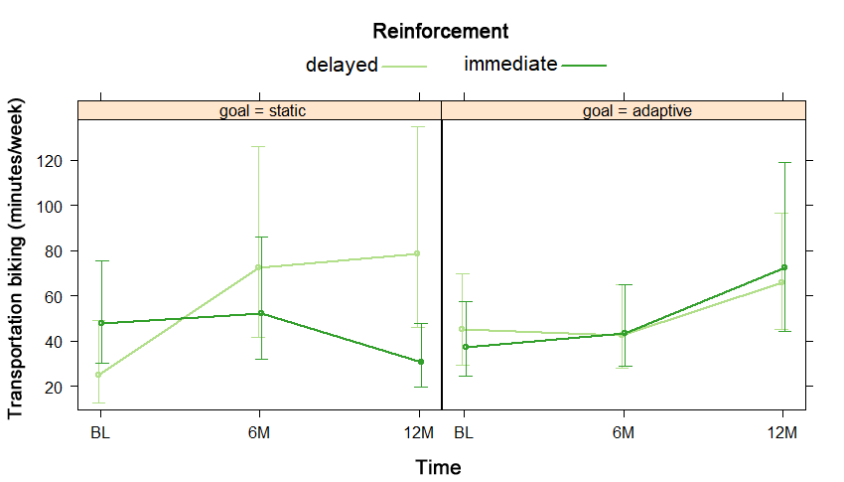
Goal by reinforcement by time interaction in negative binomial count model 3 for transportation biking at baseline (BL), 6 months (6M), and 12 months (12M).

Conditional estimates for an NBH model testing a three-way goal by reinforcement by time interaction and nested two-way interactions for transportation biking are shown in Table 5. For the hurdle model, there was no independent effect of goal or reinforcement type on odds of reporting any (versus no) transportation biking. There was a significant three-way interaction for the hurdle model such that likelihood of reporting any biking varied over time by reinforcement and goal type, as shown in Figure 8. Among participants who endorsed transportation biking in the count model, the mean reported time was 53.31 minutes/week (95% CI 30.19-94.14). There were no significant independent effects of goal or reinforcement type on transportation biking minutes/week. There was a significant three-way interaction for the count model, such that the effect of reinforcement differed by goal and time (RR=3.79, 95% CI 1.71-8.40). As shown in Figure 9, participants with delayed reinforcement and static goals reported the greatest increase in transportation biking from baseline to 12 months, whereas those with immediate reinforcement and static goals were the only group to report a decrease in transportation biking time. Immediate reinforcement had the opposite effect from 6 to 12 months among participants with adaptive goals.

**Table 5:** Negative binomial hurdle model examining goal by reinforcement by time (model 3) for transportation biking.

	Zero hurdle model		Count model	
Parameter^a^	OR^b,c^ (95% CI)	*P* value	RR^d^ (95% CI)	*P* value
Intercept	0.016 (0.00002-0.001)	<.001****	53.31 (30.19-94.14)	<.001****
Socioeconomic status block (high)	1.32 (0.40-4.36)	.651	0.74 (0.52-1.06)	.104
Walkability block (high)	0.85 (0.26-2.79)	.794	1.30 (0.91-1.85)	.151
Goal (adaptive)	3.87 (0.57-26.16)	.165	0.96 (0.56-1.66)	.895
Reinforcement (immediate)	2.25 (0.30-16.93)	.432	0.82 (0.45-1.47)	.496
Time: linear	10.22 (1.27-82.43)	.029**	2.26 (1.38-3.69)	.001***
Time: quadratic	0.39 (0.08-1.95)	.250	0.67 (0.44-1.01)	.055*
Goal by time: linear	0.21 (0.02-2.05)	.182	0.58 (0.32-1.06)	.076*
Goal by time: quadratic	2.68 (0.42-17.12)	.297	1.84 (1.05-3.23)	.034**
Reinforcement by time: linear	0.17 (0.02-1.85)	.147	0.32 (0.18-0.59)	<.001****
Reinforcement by time: quadratic	5.10 (0.67-39.07)	.117	1.16 (0.68-1.99)	.590
Goal by reinforcement	0.36 (0.03-4.69)	.436	1.19 (0.57-2.49)	.637
Goal by reinforcement by time: linear	1.91 (0.12-29.97)	.645	3.79 (1.71-8.40)	.001***
Goal by reinforcement by time: Quadratic	0.08 (0.01-0.99)	.049**	0.82 (0.39-1.70)	.588

^a^Referent groups for parameters are listed in parentheses.

^b^Odds ratio (OR) reflects the odds of reporting any leisure walking (versus none).

^c^OR, risk ratio (RR), and 95% CI are exponentiated coefficients of conditional estimates.

^d^RR reflects the proportional increase (values >1) or decrease (values <1) in non-zero transportation biking minutes/week associated with a one unit change in the predictor.

*P<.1.

*P<.05.

**P<.01.

***P<.001.

## Discussion

### Principal Findings

This study reported the secondary outcomes of the WalkIT Arizona trial, a 12-month mHealth intervention combining goal setting (adaptive versus static goals) with financial reinforcement (immediate versus delayed) to increase PA among insufficiently active adults. Analyses examined differences in self-reported PA by intervention group, activity type (walking, biking), and activity domain (transportation, leisure). It is notable that only a small subset of participants (no more than 8% of the sample at any time point) reported biking, which was limited to the transportation domain. While walking was an activity readily accessible to all participants, biking required additional equipment and skills, including perceived comfort and safety while riding. Biking for transportation likely also required additional planning with regard to route, weather, and storage. For these reasons, we have focused our discussion on walking, as these findings represent a larger proportion of the study sample and are more likely to be generalizable.

All intervention groups reported greater time walking at 12 months relative to baseline for both leisure and transportation, with differences in the trajectory of walking time observed by group and domain. Closer examination of effects using NBH count models indicated differences in duration of walking time by domain: the independent effect of goal (model 1), a reinforcement by time interaction (models 2 and 3), and a three-way goal by reinforcement by time interaction (model 3) were only significant for leisure walking. Time was the only significant independent factor contributing to the count model for reported transportation walking in both complete case and multiple imputation analyses. Prior studies that have shown this effect for goal type [12,13] have utilized objective data to capture MVPA. This study is the first to report differential effects of adaptive versus static goals for self-reported walking domains.

Although the WalkIT Arizona intervention did not target any specific domain of activity, differential effects were observed for transportation and leisure walking, and our hypotheses regarding similar intervention effects across leisure and transportation domains were not supported. However, these findings show that adaptive goals alone were similarly effective to static goals at increasing reported leisure and transportation activities over time. Immediate reinforcement alone or combined with goal setting were more effective than delayed reinforcement at increasing leisure walking at 12 months but not transportation-related walking. It is possible that immediate financial reinforcement is a stronger intervention stimulus to promote leisure walking than delayed reinforcement, but not a strong enough stimulus to overcome barriers (eg, low walkability) to adopting transportation walking. While we adjusted models for block-level walkability, we did not account for distance between participants’ home and work, or walkability surrounding their workplace. These factors may further explain some of our findings, as this study occurred in a large, sprawling metropolitan area. These results support the premise that individual-level PA interventions are domain- and context specific and could be helpful in guiding further multilevel intervention refinement.

It is interesting to compare these findings with primary study outcomes that utilized accelerometer-measured MVPA. In primary analyses, a main effect of goal type was significant such that those with adaptive goals had a greater probability of initiating any MVPA bout minutes/day (versus none), whereas a main effect of reinforcement was significant such that immediate reinforcement was more successful at increasing total MVPA bout minutes/day. Interactions between goal type and reinforcement timing on MVPA bout minutes/day indicated that the group with adaptive goals combined with immediate reinforcement outperformed other groups, except for the group with static goals combined with immediate reinforcement. Analyses with self-reported data also indicated differences between hurdle and count models, although these findings were less robust. For hurdle models, a goal by time effect was observed only for leisure walking and was nonsignificant. Count models showed a main effect for goal type favoring static goals and a main effect for reinforcement timing favoring immediate reinforcement, as well as a goal by reinforcement by time interaction supporting static or adaptive goals combined with immediate reinforcement. These results were also only significant for leisure walking. There were no significant effects of intervention parameters for reported minutes of transportation walking; an observed reinforcement by time interaction was no longer significant in multiple imputation analyses.

Our registered secondary aim referred to self-reported PA as measured by the IPAQ but was not specific to walking or cycling. Although self-reported PA may be less accurate than objective data, the examination of domain-specific PA (eg, transportation versus leisure walking) provides a better conceptual alignment and allows for a more comprehensive understanding of participant behavior within a walking intervention, which may be useful in guiding intervention refinement. The WalkIT Arizona intervention maintained a broad focus on increasing ambulatory activities at a moderate intensity or greater. While there was little difference in the proportion of participants who endorsed any walking for leisure versus transportation, reported duration of walking increased significantly more for leisure walking than transportation walking at 12 months. These findings are consistent with prior studies indicating the prevalence and correlates of leisure walking differ from those of transportation walking [7,8,21,22]. Some studies have suggested that gender may impact the domain of activity, with women being more likely to report walking for leisure and men to report walking for transportation, despite no gender difference observed in total walking for any purpose [6]. The fact that 64.5% (330/512) of the current sample were women may have contributed to a greater increase in leisure walking compared with transportation walking. Other research has noted that leisure walking tends to increase with age [22], while attributes of the built environment (eg, walkability) have consistently been shown to impact transportation walking more than leisure walking. These latter points may suggest greater flexibility with leisure walking, suggesting this domain may be more receptive to change with these individual-level intervention components. As such, an mHealth intervention utilizing goals and reinforcement, without intervening on walking destinations and access to transit, was less effective at increasing transportation PA.

### Study Limitations

The reported findings should be considered in the context of several limitations. As this study reported on secondary outcomes, the WalkIT Arizona trial was powered to detect effects using accelerometer data and not self-reported PA, which has greater variability. We elected to examine main effects of parameter by time using two-way interactions in models 1 and 2, as these models had greater power than model 3, which included a third interaction term. Lack of power may have also contributed to differences in findings between these analyses and the primary outcomes. Concerns have also been raised regarding the accuracy and sensitivity of self-reported PA, as correlations with objective measures tend to be low to moderate [11]. However, self-reported PA offered a unique opportunity to capture the context of participants walking activity, an aspect not readily accessible through objective measures in this study. Notably, self-reported PA was collected at only three time points that inquired about behavior over the previous week and may not necessarily have reflected more nuanced variability in PA over the course of the intervention. We used an intent-to-treat approach to preserve randomization and performed a sensitivity analysis comparing complete cases to multiple imputation. Results from complete case analysis and multiple imputation analyses were more consistent for leisure walking than for transportation walking.

### Conclusion

This is the first study to report differential effects of adaptive versus static goals and immediate versus delayed reinforcement for self-reported walking by domain. Despite limited power for these secondary analyses, the results support the premise that individual-level PA interventions are domain- and context-specific. This information may be helpful in guiding intervention refinement and increasing generalizability to other populations.

## Appendix

Multimedia Appendix 1 Multiple imputation negative binomial hurdle model examining goal x time interaction (model 1) for leisure walking.

Multimedia Appendix 2 Multiple imputation negative binomial hurdle model examining reinforcement x time interaction (model 2) for leisure walking.

Multimedia Appendix 3 Multiple imputation negative binomial hurdle model examining goal x reinforcement x time interaction (model 3) for leisure walking.

Multimedia Appendix 4 Multiple imputation negative binomial hurdle model examining goal x time Interaction (model 1) for transportation walking.

Multimedia Appendix 5 Multiple imputation negative binomial hurdle model examining reinforcement x time interaction (model 2) for transportation walking.

Multimedia Appendix 6 Multiple imputation negative binomial hurdle model examining goal x reinforcement x time interaction (model 3) for transportation walking.

Multimedia Appendix 7 Negative binomial hurdle model examining goal x time interaction (model 1) for leisure walking.

Multimedia Appendix 8 Negative binomial hurdle model examining reinforcement x time interaction (model 2) for leisure walking.

Multimedia Appendix 9 Negative binomial hurdle model examining goal x time interaction (model 1) for transportation walking.

Multimedia Appendix 10 Negative binomial hurdle model examining reinforcement x time interaction (model 2) for transportation walking.

Multimedia Appendix 11 Negative binomial hurdle model examining goal x time interaction (model 1) for transportation biking.

Multimedia Appendix 12 Negative binomial hurdle model examining reinforcement x time interaction (model 2) for transportation biking.

Multimedia Appendix 13 CONSORT-EHEALTH checklist v1.6.1.

## Abbreviations

glmmTMB: generalized linear mixed models using template model builder
IPAQ: International Physical Activity Questionnaire
mHealth: mobile health
MVPA: moderate-to-vigorous physical activity
NBH: negative binomial hurdle
PA: physical activity
RR: risk ratio
