# Supplementary material for: Effects of Goal Type and Reinforcement Type on Self-Reported Domain-Specific Walking Among Inactive Adults: 2×2 Factorial Randomized Controlled Trial
Source: JMIR Form Res. 2020 Dec 4;4(12):e19863. doi: 10.2196/19863 (PMC7748953; doi:10.2196/19863)
Supplement: Multimedia Appendix 1 [file formative_v4i12e19863_app1.docx]

Multimedia Appendix 1

Multiple imputation negative binomial hurdle model examining goal x time interaction (model 1) for leisure walking

|  | Zero hurdle model | | Count model | |
| --- | --- | --- | --- | --- |
| Parameter^a^ | OR^b,d^ (95% CI)^d^ | P value | RR^c,d^ (95% CI)^d^ | P value |
| Intercept | 2.80 (2.01, 3.91) | <.001*** | 93.69 (81.06, 108.29) | <.001*** |
| SES block (high) | 0.86 (0.65, 1.14) | .287 | 0.92 (0.80, 1.05) | .198 |
| Walkability block (high) | 0.94 (1.24, 0.71) | .657 | 1.02 (0.90, 1.17) | .735 |
| Reinforcement (immediate) | 0.89 (0.67, 1.17) | .391 | 1.07 (0.94, 1.22) | .331 |
| Goal (adaptive) | 1.15 (0.87, 1.51) | .340 | 0.86 (0.75, 0.98) | .024* |
| Time: linear | 1.48 (1.12, 1.96) | .007** | 1.29 (1.14, 1.47) | <.001*** |
| Time: quadratic | 0.75 (0.56, 1.00) | .059. | 0.79 (0.70, 0.89) | <.001*** |
| Goal by time: linear | 1.44 (0.96, 2.15) | .076 | 0.94 (0.79, 1.12) | .476 |
| Goal by time: quadratic | 0.89 (0.59, 1.34) | .569 | 1.12 (0.95, 1.33) | .173 |

^a^Referent groups for parameters are listed in parentheses.

^b^Odds ratio (OR) reflects the odds of reporting any leisure walking (versus none).

^c^Risk Ratio (RR) reflects the proportional increase (values >1) or decrease (values <1) in non-zero leisure walking minutes/week associated with a one unit change in the predictor.

^d^OR, RR, and 95% CI are exponentiated coefficients of conditional estimates.

.*P*<.1.

**P*<.05.

***P*<.01.

****P*<.001.
