# Supplementary material for: Effects of Goal Type and Reinforcement Type on Self-Reported Domain-Specific Walking Among Inactive Adults: 2×2 Factorial Randomized Controlled Trial
Source: JMIR Form Res. 2020 Dec 4;4(12):e19863. doi: 10.2196/19863 (PMC7748953; doi:10.2196/19863)
Supplement: Multimedia Appendix 2 [file formative_v4i12e19863_app2.docx]

Multimedia Appendix 2

Multiple imputation negative binomial hurdle model examining reinforcement x time interaction (model 2) for leisure walking

|  | Zero hurdle model | | Count model | |
| --- | --- | --- | --- | --- |
| Parameter^a^ | OR^b,d^ (95% CI)^d^ | P value | RR^c,d^ (95% CI)^d^ | P value |
| Intercept | 2.84 (2.04, 3.96) | <.001*** | 95.19 (81.90, 110.63) | <.001*** |
| SES block (high) | 0.86 (0.65, 1.13) | .286 | 0.90 (0.79, 1.03) | .133 |
| Walkability block (high) | 0.94 (0.71, 1.24) | .655 | 1.02 (0.89, 1.16) | .807 |
| Goal (adaptive) | 1.12 (0.85, 1.48) | .418 | 0.86 (0.75, 0.98) | .025* |
| Reinforcement (immediate) | 0.88 (0.66, 1.16) | .357 | 1.05 (0.92, 1.20) | .445 |
| Time: linear | 1.94 (1.45, 2.60) | <.001*** | 1.14 (1.01, 1.30) | .033* |
| Time: quadratic | 0.69 (0.51, 0.94) | .018* | 0.93 (0.83, 1.05) | .221 |
| Reinforcement by time: linear | 0.84 (0.56, 1.25) | .387 | 1.21 (1.01, 1.44) | .037* |
| Reinforcement by time: quadratic | 1.04 (0.68, 1.57) | .863 | 0.80 (0.68, 0.95) | .011* |

^a^Referent groups for parameters are listed in parentheses.

^b^Odds ratio (OR) reflects the odds of reporting any leisure walking (versus none).

^c^Risk Ratio (RR) reflects the proportional increase (values >1) or decrease (values <1) in non-zero leisure walking minutes/week associated with a one unit change in the predictor.

^d^OR, RR, and 95% CI are exponentiated coefficients of conditional estimates.

.*P*<.1.

**P*<.05.

***P*<.01.

****P*<.001.
