# Supplementary material for: Effects of Goal Type and Reinforcement Type on Self-Reported Domain-Specific Walking Among Inactive Adults: 2×2 Factorial Randomized Controlled Trial
Source: JMIR Form Res. 2020 Dec 4;4(12):e19863. doi: 10.2196/19863 (PMC7748953; doi:10.2196/19863)
Supplement: Multimedia Appendix 3 [file formative_v4i12e19863_app3.docx]

Multimedia Appendix 3

Multiple imputation negative binomial hurdle model examining goal x reinforcement x time interaction (model 3) for leisure walking

|  | Zero hurdle model | | | Count model | | |
| --- | --- | --- | --- | --- | --- | --- |
| Parameter^a^ | | OR^b,d^ (95% CI)^d^ | P value | | RR^c,d^ (95% CI)^d^ | P value |
| Intercept | | 2.31 (1.41, 3.79) | <.001*** | | 94.32 (80.02, 111.16) | <.001*** |
| SES block (high) | | 0.86 (0.65, 1.14) | .300 | | 0.91 (0.80, 1.04) | .161 |
| Walkability block (high) | | 0.95 (0.72, 1.25) | .710 | | 1.02 (0.89, 1.16) | .800 |
| Goal (adaptive) | | 1.09 (0.72, 1.65) | .688 | | 0.86 (0.71, 1.03) | .105 |
| Reinforcement (immediate) | | 0.92 (0.62, 1.35) | .659 | | 1.04 (0.86, 1.26) | .678 |
| Time: linear | | 1.35 (0.89, 2.04) | .232 | | 1.08 (0.91, 1.29) | .374 |
| Time: quadratic | | 0.76 (0.50, 1.16) | .222 | | 0.94 (0.79, 1.11) | .451 |
| Goal by time: linear | | 1.78 (0.98, 3.24) | .077 . | | 1.12 (0.88, 1.43) | .365 |
| Goal by time: quadratic | | 0.90 (0.49, 1.64) | .730 | | 0.98 (0.77, 1.23) | .833 |
| Reinforcement by time: linear | | 1.07 (0.61, 1.88) | .806 | | 1.46 (1.14, 1.86) | .003** |
| Reinforcement by time: quadratic | | 1.05 (0.59, 1.89) | .857 | | 0.70 (0.55, 0.88) | .003** |
| Goal by reinforcement | | 0.95 (0.54, 1.67) | .848 | | 1.02 (0.78, 1.33) | .885 |
| Goal by reinforcement by time: linear | | 0.61 (0.27, 1.38) | .238 | | 0.69 (0.48, 0.97) | .034* |
| Goal by reinforcement by time: quadratic | | 0.96 (0.42, 2.20) | .916 | | 1.34 (0.96, 1.87) | .086 . |

^a^Referent groups for parameters are listed in parentheses.

^b^Odds ratio (OR) reflects the odds of reporting any leisure walking (versus none).

^c^Risk Ratio (RR) reflects the proportional increase (values >1) or decrease (values <1) in non-zero leisure walking minutes/week associated with a one unit change in the predictor.

^d^OR, RR, and 95% CI are exponentiated coefficients of conditional estimates.

.*P*<.1.

**P*<.05.

***P*<.01.

****P*<.001.
