# Supplementary material for: Effects of Goal Type and Reinforcement Type on Self-Reported Domain-Specific Walking Among Inactive Adults: 2×2 Factorial Randomized Controlled Trial
Source: JMIR Form Res. 2020 Dec 4;4(12):e19863. doi: 10.2196/19863 (PMC7748953; doi:10.2196/19863)
Supplement: Multimedia Appendix 4 [file formative_v4i12e19863_app4.docx]

Multimedia Appendix 4

Multiple imputation negative binomial hurdle model examining goal x time Interaction (model 1) for transportation walking

|  | Zero hurdle model | | Count model | |
| --- | --- | --- | --- | --- |
| Parameter^a^ | OR^b,d^ (95% CI)^d^ | P value | RR^c,d^ (95% CI)^d^ | P value |
| Intercept | 2.29 (1.55, 3.36) | <.001*** | 78.71 (65.27, 94.92) | <.001*** |
| SES block (high) | 0.87 (0.61, 1.23) | .444 | 0.73 (0.62, 0.86) | <.001*** |
| Walkability block (high) | 1.53 (1.08, 2.15) | .034* | 1.05 (0.89, 1.24) | .547 |
| Reinforcement (immediate) | 0.99 (0.71, 1.38) | .949 | 1.00 (0.85, 1.17) | .971 |
| Goal (adaptive) | 0.84 (0.60, 1.16) | .286 | 0.98 (0.83, 1.15) | .761 |
| Time: linear | 1.34 (0.97, 1.83) | .170 | 1.13 (0.99, 1.29) | .064. |
| Time: quadratic | 0.68 (0.49, 0.93) | .022* | 0.76 (0.67, 0.86) | <.001*** |
| Goal by time: linear | 1.08 (0.71, 1.64) | .714 | 1.10 (0.91, 1.33) | .323 |
| Goal by time: quadratic | 1.11 (0.72, 1.71) | .642 | 1.10 (0.92 (1.33) | .301 |

^a^Referent groups for parameters are listed in parentheses.

^b^Odds ratio (OR) reflects the odds of reporting any leisure walking (versus none).

^c^Risk Ratio (RR) reflects the proportional increase (values >1) or decrease (values <1) in non-zero transportation walking minutes/week associated with a one unit change in the predictor.

^d^OR, RR, and 95% CI are exponentiated coefficients of conditional estimates.

.*P*<.1.

**P*<.05.

***P*<.01.

****P*<.001.
