# Supplementary material for: Effects of Goal Type and Reinforcement Type on Self-Reported Domain-Specific Walking Among Inactive Adults: 2×2 Factorial Randomized Controlled Trial
Source: JMIR Form Res. 2020 Dec 4;4(12):e19863. doi: 10.2196/19863 (PMC7748953; doi:10.2196/19863)
Supplement: Multimedia Appendix 5 [file formative_v4i12e19863_app5.docx]

Multimedia Appendix 5

Multiple imputation negative binomial hurdle model examining reinforcement x time interaction (model 2) for transportation walking

|  | Zero hurdle model | | Count model | |
| --- | --- | --- | --- | --- |
| Parameter^a^ | OR^b,d^ (95% CI)^d^ | P value | RR^c,d^ (95% CI)^d^ | P value |
| Intercept | 2.29 (1.55, 3.36) | <.001*** | 78.91 (65.45, 95.15) | <.001*** |
| SES block (high) | 0.82 (0.59, 1.15) | .252 | 0.73 (0.62, 0.85) | <.001*** |
| Walkability block (high) | 1.59 (1.14, 2.22) | .006** | 1.05 (0.89, 1.24) | .533 |
| Goal (adaptive) | 0.84 (0.60, 1.16) | .287 | 0.98 (0.83, 1.15) | .779 |
| Reinforcement (immediate) | 1.00 (0.72, 1.38) | .977 | 0.99 (0.84, 1.17) | .909 |
| Time: linear | 1.53 (1.13, 2.07) | .007** | 1.09 (0.95, 1.24) | .222 |
| Time: quadratic | 0.69 (0.51, 0.94) | .019* | 0.81 (0.71, 0.92) | .002** |
| Reinforcement by time: linear | 0.94 (0.62, 1.43) | .772 | 1.19 (0.99, 1.44) | .072 . |
| Reinforcement by time: quadratic | 1.02 (0.66, 1.57) | .921 | 0.98 (0.82, 1.18) | .822 |

^a^Referent groups for parameters are listed in parentheses.

^b^Odds ratio (OR) reflects the odds of reporting any leisure walking (versus none).

^c^Risk Ratio (RR) reflects the proportional increase (values >1) or decrease (values <1) in non-zero transportation walking minutes/week associated with a one unit change in the predictor.

^d^OR, RR, and 95% CI are exponentiated coefficients of conditional estimates.

.*P*<.1.

**P*<.05.

***P*<.01.

****P*<.001.
