# Supplementary material for: Effects of Goal Type and Reinforcement Type on Self-Reported Domain-Specific Walking Among Inactive Adults: 2×2 Factorial Randomized Controlled Trial
Source: JMIR Form Res. 2020 Dec 4;4(12):e19863. doi: 10.2196/19863 (PMC7748953; doi:10.2196/19863)
Supplement: Multimedia Appendix 6 [file formative_v4i12e19863_app6.docx]

Multimedia Appendix 6

Multiple imputation negative binomial hurdle model examining goal x reinforcement x time interaction (model 3) for transportation walking

|  | Zero hurdle model | | | Count model | |
| --- | --- | --- | --- | --- | --- |
| Parameter^a^ | | OR^b,d^ (95% CI)^d^ | P value | RR^c,d^ (95% CI)^d^ | P value |
| Intercept | | 2.49 (1.63, 3.80) | <.001*** | 77.67 (63.41, 95.14) | <.001*** |
| SES block (high) | | 0.82 (0.59, 1.15) | .252 | 0.72 (0.62, 0.85) | <.001*** |
| Walkability block (high) | | 1.59 (1.14, 2.22) | .006** | 1.05 (0.89, 1.24) | .539 |
| Goal (adaptive) | | 0.71 (0.44, 1.14) | .154 | 1.01 (0.81, 1.28) | .907 |
| Reinforcement (immediate) | | 0.85 (0.53, 1.36) | .489 | 1.02 (0.82, 1.29) | .834 |
| Time: linear | | 1.33 (0.86, 2.04) | .201 | 1.04 (0.86, 1.24) | .694 |
| Time: quadratic | | 0.74 (0.48, 1.15) | .186 | 0.78 (0.65, 0.93) | .009** |
| Goal by time: linear | | 1.33 (0.73, 2.42) | .351 | 1.11 (0.84, 1.45) | .465 |
| Goal by time: quadratic | | 0.86 (0.47, 1.60) | .637 | 1.07 (0.82, 1.39) | .623 |
| Reinforcement by time: linear | | 1.16 (0.64, 2.11) | .627 | 1.21 (0.92, 1.57) | .164 |
| Reinforcement by time: quadratic | | 0.80 (0.43, 1.48) | .478 | 0.95 (0.73, 1.22) | .668 |
| Goal by reinforcement | | 1.38 (0.71, 2.68) | .339 | 0.93 (0.67, 1.29) | .667 |
| Goal by reinforcement by time: linear | | 0.66 (0.28, 1.53) | .332 | 0.97 (0.66, 1.41) | .870 |
| Goal by reinforcement by time: quadratic | | 1.64 (0.69, 3.91) | .268 | 1.07 (0.74, 1.55) | .703 |

^a^Referent groups for parameters are listed in parentheses.

^b^Odds ratio (OR) reflects the odds of reporting any leisure walking (versus none).

^c^Risk Ratio (RR) reflects the proportional increase (values >1) or decrease (values <1) in non-zero transportation walking minutes/week associated with a one unit change in the predictor.

^d^OR, RR, and 95% CI are exponentiated coefficients of conditional estimates.

.*P*<.1.

**P*<.05.

***P*<.01.

****P*<.001.
