# Supplementary material for: Effects of Goal Type and Reinforcement Type on Self-Reported Domain-Specific Walking Among Inactive Adults: 2×2 Factorial Randomized Controlled Trial
Source: JMIR Form Res. 2020 Dec 4;4(12):e19863. doi: 10.2196/19863 (PMC7748953; doi:10.2196/19863)
Supplement: Multimedia Appendix 7 [file formative_v4i12e19863_app7.docx]

Multimedia Appendix 7

Negative binomial hurdle model examining goal x time interaction (model 1) for leisure walking

|  | Zero hurdle model | | Count model | |
| --- | --- | --- | --- | --- |
| Parameter^a^ | OR^b,d^ (95% CI)^d^ | P value | RR^c,d^ (95% CI)^d^ | P value |
| Intercept | 3.33 (2.33, 4.76) | <.001*** | 96.60 (82.50, 113.11) | <.001*** |
| SES block (high) | 0.82 (0.60, 1.11) | .203 | 0.90 (0.78, 1.03) | .131 |
| Walkability block (high) | 0.94 (0.69, 1.28) | .711 | 1.04 (0.90, 1.19) | .588 |
| Reinforcement (immediate) | 0.93 (0.68, 1.27) | .270 | 1.08 (0.94, 1.24) | .290 |
| Goal (adaptive) | 2.94 (0.87, 1.64) | .642 | 0.83 (0.72, 0.95) | .009** |
| Time: linear | 1.85 (1.35, 2.56) | <.001*** | 1.36 (1.20, 1.54) | <.001*** |
| Time: quadratic | 0.75 (0.54, 1.03) | .079 | 0.78 (0.69, 0.88) | <.001*** |
| Goal by time: linear | 1.49 (0.95, 2.38) | .080 . | 0.91 (0.76, 1.09) | .301 |
| Goal by time: quadratic | 0.81 (0.50, 1.30) | .382 | 1.13 (0.95, 1.34) | .182 |

^a^Referent groups for parameters are listed in parentheses.

^b^Odds ratio (OR) reflects the odds of reporting any leisure walking (versus none).

^c^Risk Ratio (RR) reflects the proportional increase (values >1) or decrease (values <1) in non-zero leisure walking minutes/week associated with a one unit change in the predictor.

^d^OR, RR, and 95% CI are exponentiated coefficients of conditional estimates.

.*P*<.1.

**P*<.05.

***P*<.01.

****P*<.001.
