# Supplementary material for: Effects of Goal Type and Reinforcement Type on Self-Reported Domain-Specific Walking Among Inactive Adults: 2×2 Factorial Randomized Controlled Trial
Source: JMIR Form Res. 2020 Dec 4;4(12):e19863. doi: 10.2196/19863 (PMC7748953; doi:10.2196/19863)
Supplement: Multimedia Appendix 8 [file formative_v4i12e19863_app8.docx]

Multimedia Appendix 8

Negative binomial hurdle model examining reinforcement x time interaction (model 2) for leisure walking

|  | Zero hurdle model | | Count model | |
| --- | --- | --- | --- | --- |
| Parameter^a^ | OR^b,d^ (95% CI)^d^ | P value | RR^c,d^ (95% CI)^d^ | P value |
| Intercept | 3.45 (2.38, 5.00) | <.001*** | 97.49 (83.39, 113.98) | <.001*** |
| SES block (high) | 0.82 (0.60, 1.11) | .203 | 0.88 (0.77, 1.01) | .075 . |
| Walkability block (high) | 0.94 (0.69, 1.28) | .711 | 1.03 (0.90, 1.18) | .655 |
| Goal (adaptive) | 1.11 (0.81, 1.52) | .507 | 0.84 (0.73, 0.96) | .012* |
| Reinforcement (immediate) | 0.91 (0.66, 1.25) | .545 | 1.08 (0.94, 1.24) | .282 |
| Time: linear | 2.38 (1.72, 3.33) | <.001*** | 1.18 (1.04, 1.34) | .010* |
| Time: quadratic | 0.66 (0.47, 0.93) | .017* | 0.93 (0.82, 1.05) | .226 |
| Reinforcement by time: linear | 0.88 (0.56, 1.39) | .603 | 1.22 (1.02, 1.46) | .031* |
| Reinforcement by time: quadratic | 1.03 (0.64, 1.67) | .888 | 0.79 (0.66, 0.94) | .008** |

^a^Referent groups for parameters are listed in parentheses.

^b^Odds ratio (OR) reflects the odds of reporting any leisure walking (versus none).

^c^Risk Ratio (RR) reflects the proportional increase (values >1) or decrease (values <1) in non-zero leisure walking minutes/week associated with a one unit change in the predictor.

^d^OR, RR, and 95% CI are exponentiated coefficients of conditional estimates.

.*P*<.1.

**P*<.05.

***P*<.01.

****P*<.001.
