# Supplementary material for: Effects of Goal Type and Reinforcement Type on Self-Reported Domain-Specific Walking Among Inactive Adults: 2×2 Factorial Randomized Controlled Trial
Source: JMIR Form Res. 2020 Dec 4;4(12):e19863. doi: 10.2196/19863 (PMC7748953; doi:10.2196/19863)
Supplement: Multimedia Appendix 9 [file formative_v4i12e19863_app9.docx]

Multimedia Appendix 9

Negative binomial hurdle model examining goal x time interaction (model 1) for transportation walking

|  | Zero hurdle model | | Count model | |
| --- | --- | --- | --- | --- |
| Parameter^a^ | OR^b,d^ (95% CI)^d^ | P value | RR^c,d^ (95% CI)^d^ | P value |
| Intercept | 2.78 (1.72, 4.35) | <.001*** | 82.46 (67.52, 100.71) | <.001*** |
| SES block (high) | 0.79 (0.54, 1.18) | .248 | 0.70 (0.59, 0.83) | <.001*** |
| Walkability block (high) | 1.82 (1.22, 2.70) | .003** | 1.03 (0.86, 1.22) | .771 |
| Reinforcement (immediate) | 0.97 (0.65, 1.43) | .864 | 0.99 (0.83, 1.18) | .901 |
| Goal (adaptive) | 0.77 (0.52, 1.14) | .190 | 0.97 (0.82, 1.15) | .725 |
| Time: linear | 1.79 (1.27, 2.50) | <.001*** | 1.18 (1.03, 1.34) | .018* |
| Time: quadratic | 0.67 (0.47, 0.95) | .028* | 0.74 (0.64, 0.84) | <.001*** |
| Goal by time: linear | 1.02 (0.63, 1.64) | .946 | 1.12 (0.92, 1.35) | .267 |
| Goal by time: quadratic | 1.06 (0.65, 1.75) | .815 | 1.15 (0.95, 1.40) | .156 |

^a^Referent groups for parameters are listed in parentheses.

^b^Odds ratio (OR) reflects the odds of reporting any leisure walking (versus none).

^c^Risk Ratio (RR) reflects the proportional increase (values >1) or decrease (values <1) in non-zero transportation walking minutes/week associated with a one unit change in the predictor.

^d^OR, RR, and 95% CI are exponentiated coefficients of conditional estimates.

.*P*<.1.

**P*<.05.

***P*<.01.

****P*<.001.
