# Supplementary material for: Effects of Goal Type and Reinforcement Type on Self-Reported Domain-Specific Walking Among Inactive Adults: 2×2 Factorial Randomized Controlled Trial
Source: JMIR Form Res. 2020 Dec 4;4(12):e19863. doi: 10.2196/19863 (PMC7748953; doi:10.2196/19863)
Supplement: Multimedia Appendix 10 [file formative_v4i12e19863_app10.docx]

Multimedia Appendix 10

Negative binomial hurdle model examining reinforcement x time interaction (model 2) for transportation walking

|  | Zero hurdle model | | | Count model | |
| --- | --- | --- | --- | --- | --- |
| Parameter^a^ | | OR^b,d^ (95% CI)^d^ | P value | RR^c,d^ (95% CI)^d^ | P value |
| Intercept | | 2.78 (1.72, 4.35) | <.001*** | 82.27 (67.41, 100.40) | <.001*** |
| SES block (high) | | 0.79 (0.53, 1.18) | .249 | 0.70 (0.59, 0.83) | <.001*** |
| Walkability block (high) | | 1.82 (1.23, 2.70) | .003** | 1.03 (0.87, 1.23) | .732 |
| Goal (adaptive) | | 0.77 (0.52, 1.14) | .186 | 0.97 (0.82, 1.16) | .762 |
| Reinforcement (immediate) | | 0.95 (0.65, 1.43) | .826 | 0.99 (0.84, 1.18) | .937 |
| Time: linear | | 1.85 (1.33, 2.63) | <.001*** | 1.12 (0.98, 1.29) | .092. |
| Time: quadratic | | 0.67 (0.47, 0.96) | .027* | 0.80 (0.70, 0.91) | <.001*** |
| Reinforcement by time: linear | | 0.93 (0.58, 1.49) | .762 | 1.22 (1.01, 1.49) | .041* |
| Reinforcement by time: quadratic | | 1.04 (0.63, 1.72) | .871 | 0.98 (0.80, 1.19) | .811 |

^a^Referent groups for parameters are listed in parentheses.

^b^Odds ratio (OR) reflects the odds of reporting any leisure walking (versus none).

^c^Risk Ratio (RR) reflects the proportional increase (values >1) or decrease (values <1) in non-zero transportation walking minutes/week associated with a one unit change in the predictor.

^d^OR, RR, and 95% CI are exponentiated coefficients of conditional estimates.

.*P*<.1.

**P*<.05.

***P*<.01.

****P*<.001.
