# Supplementary material for: Effects of Goal Type and Reinforcement Type on Self-Reported Domain-Specific Walking Among Inactive Adults: 2×2 Factorial Randomized Controlled Trial
Source: JMIR Form Res. 2020 Dec 4;4(12):e19863. doi: 10.2196/19863 (PMC7748953; doi:10.2196/19863)
Supplement: Multimedia Appendix 11 [file formative_v4i12e19863_app11.docx]

Multimedia Appendix 11

Negative binomial hurdle model examining goal x time interaction (model 1) for transportation biking

|  | Zero hurdle model | | Count model | |
| --- | --- | --- | --- | --- |
| Parameter^a^ | OR^b,d^ (95% CI)^d^ | P value | RR^c,d^ (95% CI)^d^ | P value |
| Intercept | 0.0003 (0.0001, 0.0019) | <.001*** | 56.42 (34.55, 92.14) | <.001*** |
| SES block (high) | 1.31 (0.41, 4.20) | .651 | 0.78 (0.54, 1.12) | .172 |
| Walkability block (high) | 0.85 (0.27, 2.68) | .776 | 1.26 (0.88, 1.79) | .207 |
| Reinforcement (immediate) | 1.08 (0.34, 3.41) | .901 | 0.97 (0.67, 1.41) | .870 |
| Goal (adaptive) | 2.08 (0.63, 6.87) | .231 | 0.86 (0.60, 1.23) | .396 |
| Time: linear | 2.89 (1.12, 7.44) | .028* | 1.04 (0.75, 1.44) | .817 |
| Time: quadratic | 1.11 (0.45, 2.77) | .817 | 0.73 (0.53, 1.01) | .054 . |
| Goal by time: linear | 0.49 (0.15, 1.57) | .231 | 1.37 (0.89, 2.10) | .153 |
| Goal by time: quadratic | 0.66 (0.21, 2.06) | .478 | 1.62 (1.06, 2.47) | .025* |

^a^Referent groups for parameters are listed in parentheses.

^b^Odds ratio (OR) reflects the odds of reporting any leisure walking (versus none).

^c^Risk Ratio (RR) reflects the proportional increase (values >1) or decrease (values <1) in non-zero transportation biking minutes/week associated with a one unit change in the predictor.

^d^OR, RR, and 95% CI are exponentiated coefficients of conditional estimates.

.*P*<.1.

**P*<.05.

***P*<.01.

****P*<.001.
