# Supplementary material for: Effects of Goal Type and Reinforcement Type on Self-Reported Domain-Specific Walking Among Inactive Adults: 2×2 Factorial Randomized Controlled Trial
Source: JMIR Form Res. 2020 Dec 4;4(12):e19863. doi: 10.2196/19863 (PMC7748953; doi:10.2196/19863)
Supplement: Multimedia Appendix 12 [file formative_v4i12e19863_app12.docx]

Multimedia Appendix 12

Negative binomial hurdle model examining reinforcement x time interaction (model 2) for transportation biking

|  | Zero hurdle model | | | Count model | |
| --- | --- | --- | --- | --- | --- |
| Parameter^a^ | | OR^b,d^ (95% CI)^d^ | P value | RR^c,d^ (95% CI)^d^ | P value |
| Intercept | | 0.0003 (0.0001, 0.0019) | <.001*** | 55.15 (33.73, 90.19) | <.001*** |
| SES block (high) | | 1.30 (0.41, 4.16) | .659 | 0.76 (0.53, 1.10) | .147 |
| Walkability block (high) | | 0.85 (0.27, 2.70) | .785 | 1.26 (0.87, 1.83) | .216 |
| Goal (adaptive) | | 1.86 (0.58, 5.98) | .300 | 0.99 (0.68, 1.43) | .943 |
| Reinforcement (immediate) | | 1.19 (0.37, 3.84) | .766 | 0.85 (0.59, 1.21) | .363 |
| Time: linear | | 2.97 (1.33, 6.66) | .008** | 1.59 (1.14, 2.22) | .006** |
| Time: quadratic | | 0.83 (0.39, 1.78) | .635 | 0.94 (0.68, 1.30) | .705 |
| Reinforcement by time: linear | | 0.38 (0.13, 1.17) | .092 . | 0.62 (0.39, 0.98) | .041* |
| Reinforcement by time: quadratic | | 1.04 (0.35, 3.06) | .942 | 0.98 (0.63, 1.52) | .931 |

^a^Referent groups for parameters are listed in parentheses.

^b^Odds ratio (OR) reflects the odds of reporting any leisure walking (versus none).

^c^Risk Ratio (RR) reflects the proportional increase (values >1) or decrease (values <1) in non-zero transportation biking minutes/week associated with a one unit change in the predictor.

^d^OR, RR, and 95% CI are exponentiated coefficients of conditional estimates.

.*P*<.1.

**P*<.05.

***P*<.01.

****P*<.001.
