# Supplementary material for: Effects of Goal Type and Reinforcement Type on Self-Reported Domain-Specific Walking Among Inactive Adults: 2×2 Factorial Randomized Controlled Trial
Source: JMIR Form Res. 2020 Dec 4;4(12):e19863. doi: 10.2196/19863 (PMC7748953; doi:10.2196/19863)
Supplement: Multimedia Appendix 13 [file formative_v4i12e19863_app13.pdf]

CONSORT-EHEALTH Checklist V1.6.2 Report

Manuscript Number 19863

(based on CONSORT-EHEALTH V1.6), available at [http://tinyurl.com/consort-ehealth-v1-6].

Date completed

11/25/2020 18:47:16

by

Mindy McEntee

Effects of Goal Type and Reinforcement Type on Self-Reported Domain-Specific Walking Among Inactive Adults: 2×2 Factorial Randomized Controlled Trial

TITLE

1a-i) Identify the mode of delivery in the title

mhealth intervention' noted clearly in abstract & text

1a-ii) Non-web-based components or important co-interventions in title

"effect of goal type and reinforcement type"

1a-iii) Primary condition or target group in the title

"among inactive adults"

ABSTRACT

1b-i) Key features/functionalities/components of the intervention and comparator in the METHODS section of the ABSTRACT

"WalkIT Arizona was a 2×2 factorial trial examining the effects of goal type (adaptive versus static) and reinforcement type (immediate versus delayed) to increase moderate to vigorous physical activity (MVPA) among insufficiently active adults. The 12-month intervention combined mobile health (mHealth) technology with behavioral strategies to test scalable population-health approaches to increasing MVPA."

1b-ii) Level of human involvement in the METHODS section of the ABSTRACT

"participants in all four groups were provided with an activity monitor, which they were asked to wear on their wrist for 1 year" "The 12-month intervention combined mobile health (mHealth) technology with behavioral strategies to test scalable population-health approaches to increasing MVPA."

1b-iii) Open vs. closed, web-based (self-assessment) vs. face-to-face assessments in the METHODS section of the ABSTRACT

"Participants were screened online and via phone interview prior to attending an office visit. Inactive status was verified following a 10-day baseline period in which participants were asked to wear a wrist-worn accelerometer during their normal activities."

1b-iv) RESULTS section in abstract must contain use data

"All intervention groups reported increased walking at 12 months relative to baseline. Effects of the intervention differed by domain: a significant three-way goal by reinforcement by time interaction was observed for total minutes of leisure walking/week, whereas time was the only significant factor that contributed to transportation walking."

1b-v) CONCLUSIONS/DISCUSSION in abstract for negative trials

"This study is the first to report differential effects of adaptive versus static goals for self-reported walking by domain. Results support the premise that individual-level PA interventions are domain- and context-specific and may be helpful in guiding further intervention refinement."

INTRODUCTION

2a-i) Problem and the type of system/solution

"To date, interventions to increase PA have primarily focused on individual behavior change and had limited impact on population health [4]. Accordingly, there remains a need to develop potent interventions capable of producing an impact on a broader scale."

2a-ii) Scientific background, rationale: What is known about the (type of) system

"The WalkIT Arizona trial was designed to address this gap in the literature by studying the effects of an mHealth intervention that combined two evidence-based behavioral strategies—goal setting and positive reinforcement through the use of financial incentives—on objectively measured PA" (see intro)

Does your paper address CONSORT subitem 2b?

"We hypothesized that there would be significant main effects of goal type and reinforcement timing consistent with previous studies: those with adaptive goals would report more PA than those with static goals, and those receiving immediate reinforcement would report more PA than those receiving delayed reinforcement" (see intro)

METHODS

3a) CONSORT: Description of trial design (such as parallel, factorial) including allocation ratio

"WalkIT Arizona was a 2×2 factorial randomized trial evaluating the effects of goal setting (adaptive versus static goals) combined with financial incentives (immediate versus delayed reinforcement) to increase moderate-to-vigorous PA (MVPA) among insufficiently active adults." (see methods)

3b) CONSORT: Important changes to methods after trial commencement (such as eligibility criteria), with reasons

"Baseline was extended beyond the scheduled 10 days for some participants due to issues with the accelerometer, problems with the mobile app, nonadherence to accelerometer wear protocol, or illness."

3b-i) Bug fixes, Downtimes, Content Changes

n/a

4a) CONSORT: Eligibility criteria for participants

"Insufficiently active adults" "balanced across geographic information system–measured neighborhood walkability (high/low) and socioeconomic status (high/low) at the census block group level, with recruitment balanced across calendar months to adjust for seasonal effects." (see methods)

4a-i) Computer / Internet literacy

n/a in manuscript; details on reading proficiency and English language use cited in methods paper

4a-ii) Open vs. closed, web-based vs. face-to-face assessments:

"Self-reported data were collected at baseline and at 6, 12, 18, and 24 months. Analyses presented here were conducted following completion of the 12-month intervention."

4a-iii) Information giving during recruitment

"Participants were told they would receive one of four different PA interventions."

4b) CONSORT: Settings and locations where the data were collected

"Participants were screened online and via phone interview prior to attending an office visit" "WalkIT Arizona intervention components have been described in detail elsewhere [5]."

4b-i) Report if outcomes were (self-)assessed through online questionnaires

"Self-reported PA was assessed using sections 2, 4, and 5 of the International Physical Activity Questionnaire (IPAQ)-long form. The IPAQ was part of a larger battery of self-reported measures given at baseline, 6 months, and 12 months."

4b-ii) Report how institutional affiliations are displayed

n/a in manuscript, described in referenced methods paper "WalkIT Arizona intervention components have been described in detail elsewhere [5]."

5) CONSORT: Describe the interventions for each group with sufficient details to allow replication, including how and when they were actually administered

5-i) Mention names, credential, affiliations of the developers, sponsors, and owners

"WalkIT Arizona intervention components have been described in detail elsewhere [5]."

5-ii) Describe the history/development process

n/a in manuscript, see referenced methods paper "WalkIT Arizona intervention components have been described in detail elsewhere [5]."

5-iii) Revisions and updating

n/a; no changes to intervention components (goal setting or reinforcement types) made

5-iv) Quality assurance methods

n/a; primary outcomes utilized objective MVPA measure (accelerometer), secondary outcomes used self-report as supplementary data for better understanding of context

5-v) Ensure replicability by publishing the source code, and/or providing screenshots/screen-capture video, and/or providing flowcharts of the algorithms used

n/a; see referenced methods paper "WalkIT Arizona intervention components have been described in detail elsewhere [5]."

5-vi) Digital preservation

n/a; see referenced methods paper "WalkIT Arizona intervention components have been described in detail elsewhere [5]."

5-vii) Access

"participants could receive feedback via text message at any time on their accumulated MVPA minutes once they synced their activity monitor to the automated mHealth servers. All groups also received daily antecedent prompts using a pool of messages from our preliminary studies to evoke motivation, overcome barriers, remind about benefits, and provide other general health advice based on previous research. Text messaging was the primary communication channel between the mHealth system and participants. All feedback, goals, and reinforcement were communicated via this channel."

5-viii) Mode of delivery, features/functionalities/components of the intervention and comparator, and the theoretical framework

see referenced methods paper for details regarding adaptive goal algorithm; "Text messaging was the primary communication channel between the mHealth system and participants. All feedback, goals, and reinforcement were communicated via this channel."		
**5-ix) Describe use parameters**		
"Participants allocated to the static goal group were asked to accumulate 30 minutes or more of MVPA daily" "Participants allocated to the adaptive goal group were assigned a goal daily based on a previously tested percentile-rank<br>algorithm [12-14]."		
**5-x) Clarify the level of human involvement**		
"Text messaging was the primary communication channel between the mHealth system and participants. All feedback, goals, and reinforcement were communicated via this channel."		
**5-xi) Report any prompts/reminders used**		
"participants could receive feedback via text message at any time on their accumulated MVPA minutes once they synced their activity monitor to the automated mHealth servers. All groups also received daily antecedent prompts using a pool of messages from our preliminary studies to evoke motivation, overcome barriers, remind about benefits, and provide other general health advice based on previous research." (see methods)		
**5-xii) Describe any co-interventions (incl. training/support)**		
training/support described in referenced methods paper "WalkIT Arizona intervention components have been described in detail elsewhere [5]."		
**6a) CONSORT: Completely defined pre-specified primary and secondary outcome measures, including how and when they were assessed**		
"primary study outcomes that utilized accelerometer-measured MVPA"; "Our registered secondary aim referred to self-reported PA as measured by the IPAQ but was not specific to walking or cycling. Although self-reported PA may be less accurate than objective data, the examination of domain-specific PA (eg,<br>transportation versus leisure walking) provides a better conceptual alignment and allows for a more comprehensive understanding of participant behavior within a walking intervention, which may be useful in guiding intervention refinement."		
**6a-i) Online questionnaires: describe if they were validated for online use and apply CHERRIES items to describe how the questionnaires were designed/deployed**		
n/a; see referenced methods paper; selected measures have previously been validated		
**6a-ii) Describe whether and how "use" (including intensity of use/dosage) was defined/measured/monitored**		
n/a; daily compliance wearing device was tracked, but primary outcome (accelerometer-measured MVPA) only means of tracking "use"		
**6a-iii) Describe whether, how, and when qualitative feedback from participants was obtained**		
n/a in manuscript; qualitative feedback assessed at end of intervention via self-report survey		
**6b) CONSORT: Any changes to trial outcomes after the trial commenced, with reasons**		
"Participants were screened online and via phone interview prior to attending an office visit" "WalkIT Arizona intervention components have been described in detail elsewhere [5]."		
**7a) CONSORT: How sample size was determined**		
**7a-i) Describe whether and how expected attrition was taken into account when calculating the sample size**		
"study was powered to detect a 2.1 minute/day difference in main effects<br>and 4.2 minute/day difference in interaction effects between groups using accelerometer-measured MVPA, with a sample size of 120 participants per group."		
**7b) CONSORT: When applicable, explanation of any interim analyses and stopping guidelines**		
"primary study outcomes that utilized accelerometer-measured MVPA"; "Our registered secondary aim referred to self-reported PA as measured by the IPAQ but was not specific to walking or cycling. Although self-reported PA may be less accurate than objective data, the examination of domain-specific PA (eg,<br>transportation versus leisure walking) provides a better conceptual alignment and allows for a more comprehensive understanding of participant behavior within a walking intervention, which may be useful in guiding intervention refinement."		
**8a) CONSORT: Method used to generate the random allocation sequence**		
n/a in manuscript, see cited methods paper for details		
**8b) CONSORT: Type of randomisation; details of any restriction (such as blocking and block size)**		
n/a in manuscript, see cited methods paper for details		
**9) CONSORT: Mechanism used to implement the random allocation sequence (such as sequentially numbered containers), describing any steps taken to conceal the sequence until interventions were assigned**		
n/a in manuscript, see cited methods paper for details		
**10) CONSORT: Who generated the random allocation sequence, who enrolled participants, and who assigned participants to interventions**		
n/a in manuscript, see cited methods paper for details		
**11a) CONSORT: Blinding - If done, who was blinded after assignment to interventions (for example, participants, care providers, those assessing outcomes) and how**		
**11a-i) Specify who was blinded, and who wasn't**		
"Participants were told they would receive one of four different PA interventions." participants were not provided with details as to how these interventions differed.		
**11a-ii) Discuss e.g., whether participants knew which intervention was the "intervention of interest" and which one was the "comparator"**		
"We hypothesized that there would be significant main effects of goal type and reinforcement timing consistent with previous studies: those with adaptive goals would report more PA than those with static goals [12,13], and those receiving immediate reinforcement would report more PA than those receiving delayed reinforcement [13]."		
**11b) CONSORT: If relevant, description of the similarity of interventions**		
"Text messaging was the primary communication channel between the mHealth system and participants. All feedback, goals, and reinforcement were communicated via this channel." (see cited methods paper for further details)		
**12a) CONSORT: Statistical methods used to compare groups for primary and secondary outcomes**		
"Negative binomial hurdle (NBH) models provided a nuanced examination of differences across intervention groups by activity type and domain. NBH models tested main effects and interactions among intervention parameters (goal type,<br>reinforcement timing, time) with a random intercept allowed to vary by participant." (see methods)		
**12a-i) Imputation techniques to deal with attrition / missing values**		
"Multiple imputation was performed using the multivariate imputation by chained equations (MICE) package [17] with 12 iterations, corresponding to the percentage<br>of missing data [18]."		
**12b) CONSORT: Methods for additional analyses, such as subgroup analyses and adjusted analyses**		
"All models were adjusted for census block-level socioeconomic status and neighborhood walkability since these factors were part of the broader research design. Predictor variables were kept consistent for hurdle and count models." (see methods for more)		
RESULTS		
**13a) CONSORT: For each group, the numbers of participants who were randomly assigned, received intended treatment, and were analysed for the primary outcome**		
"Participant flow is depicted in Figure 1."		
**13b) CONSORT: For each group, losses and exclusions after randomisation, together with reasons**		
"Participant flow is depicted in Figure 1."		
**13b-i) Attrition diagram**		
"Participant flow is depicted in Figure 1."		
**14a) CONSORT: Dates defining the periods of recruitment and follow-up**		
"Insufficiently active adults aged 19 to 60 years (N=512) were randomized for participation between May 2016 and May 2018." "Participants completed a 12-month intervention followed by a 12-month observational follow-up period."		
**14a-i) Indicate if critical "secular events" fell into the study period**		
"recruitment balanced across calendar months to adjust for seasonal effects." (necessary due to hot temperatures); other personally relevant changes (e.g. moving, injury) tracked via self-report assessment as described in methods paper.		
**14b) CONSORT: Why the trial ended or was stopped (early)**		
n/a		
**15) CONSORT: A table showing baseline demographic and clinical characteristics for each group**		
"Baseline participant characteristics are displayed in Table 1." "Mean self-reported PA times by intervention group, activity type, and domain are shown in Table 2."		
**15-i) Report demographics associated with digital divide issues**		
"Baseline participant characteristics are displayed in Table 1."		
**16a) CONSORT: For each group, number of participants (denominator) included in each analysis and whether the analysis was by original assigned groups**		
**16-i) Report multiple "denominators" and provide definitions**		
"Multiple imputation was performed using the multivariate imputation by chained equations (MICE) package [17] with 12 iterations, corresponding to the percentage of missing data [18]." "Participants with at least 1 missing self-reported PA data point (121/512, 23.6%) were more likely to report living with a partner but did not significantly differ from those with complete data on any other demographic characteristic."		

**16-ii) Primary analysis should be intent-to-treat**		
"We used an intent-to-treat approach to preserve randomization and performed a sensitivity analysis comparing complete cases to multiple imputation."		
**17a) CONSORT: For each primary and secondary outcome, results for each group, and the estimated effect size and its precision (such as 95% confidence interval)**		
For all models: "Odds ratio (OR) reflects the odds of reporting any leisure walking (versus none)." "RR reflects the proportional increase (values >1) or decrease (values <1) in non-zero transportation walking minutes/week associated with a one unit change in the predictor." "OR, risk ratio (RR), and 95% CI are exponentiated coefficients of conditional estimates."		
**17a-i) Presentation of process outcomes such as metrics of use and intensity of use**		
n/a; study design did not require active "use" of mhealth system beyond syncing device.		
**17b) CONSORT: For binary outcomes, presentation of both absolute and relative effect sizes is recommended**		
n/a		
**18) CONSORT: Results of any other analyses performed, including subgroup analyses and adjusted analyses, distinguishing pre-specified from exploratory**		
"Models 1 and 2 examined two-way interactions and included the third intervention parameter (ie, goal type or reinforcement timing) as a covariate. Model 3 examined a three-way goal by reinforcement by time interaction but had less power due to the additional interaction term."		
**18-i) Subgroup analysis of comparing only users**		
"As sensitivity analysis revealed little impact of missing data, the results discussed below are for complete cases; model parameters using multiple imputation are presented in Multimedia Appendices 1 to 6. Any differences between complete case analysis and multiple imputation results are noted below."		
**19) CONSORT: All important harms or unintended effects in each group**		
n/a		
**19-i) Include privacy breaches, technical problems**		
"Baseline was extended beyond the scheduled 10 days for some participants due to issues with the accelerometer, problems with the mobile app, nonadherence to accelerometer wear protocol, or illness."		
**19-ii) Include qualitative feedback from participants or observations from staff/researchers**		
n/a; data from 24 months not complete at time of analyses		
DISCUSSION		
**20) CONSORT: Trial limitations, addressing sources of potential bias, imprecision, multiplicity of analyses**		
**20-i) Typical limitations in ehealth trials**		
"The reported findings should be considered in the context of several limitations. As this study reported on secondary outcomes, the WalkIT Arizona trial was powered to detect effects using accelerometer data and not self-reported PA, which has greater variability." (see discussion)		
**21) CONSORT: Generalisability (external validity, applicability) of the trial findings**		
**21-i) Generalizability to other populations**		
"Notably, self-reported PA was collected at only three time points that inquired about behavior over the previous week and may not necessarily have reflected more nuanced variability in PA over the course of the intervention."		
**21-ii) Discuss if there were elements in the RCT that would be different in a routine application setting**		
n/a; study was conducted in free-living environment		
**22) CONSORT: Interpretation consistent with results, balancing benefits and harms, and considering other relevant evidence**		
**22-i) Restate study questions and summarize the answers suggested by the data, starting with primary outcomes and process outcomes (use)**		
"Although the WalkIT Arizona intervention did not target any specific domain of activity, differential effects were observed for transportation and leisure walking, and our hypotheses regarding similar intervention effects across leisure and transportation domains were not supported." (see discussion)		
**22-ii) Highlight unanswered new questions, suggest future research**		
"These latter points may suggest greater flexibility with leisure walking, suggesting this domain may be more receptive to change with these individual-level intervention components." (see discussion)		
Other information		
**23) CONSORT: Registration number and name of trial registry**		
"This work was supported by the National Cancer Institute at the National Institutes of Health (R01CA198915)." see also: https://clinicaltrials.gov/ct2/show/NCT02717663		
**24) CONSORT: Where the full trial protocol can be accessed, if available**		
n/a; see referenced methods paper for additional details		
**25) CONSORT: Sources of funding and other support (such as supply of drugs), role of funders**		
"This work was supported by the National Cancer Institute at the National Institutes of Health (R01CA198915)." see also: https://clinicaltrials.gov/ct2/show/NCT02717663		
**X26-i) Comment on ethics committee approval**		
"This study was approved by the local institutional review board; further study details are published elsewhere [5]."		
**x26-ii) Outline informed consent procedures**		
n/a in manuscript, see cited methods paper "further study details are published elsewhere [5]."		
**X26-iii) Safety and security procedures**		
n/a; see cited methods paper and primary outcomes paper		
**X27-i) State the relation of the study team towards the system being evaluated**		
"none declared."		
